# MAS NMR on a Red/Far-Red Photochromic Cyanobacteriochrome All2699 from *Nostoc*

**DOI:** 10.3390/ijms20153656

**Published:** 2019-07-26

**Authors:** Qian-Zhao Xu, Pavlo Bielytskyi, James Otis, Christina Lang, Jon Hughes, Kai-Hong Zhao, Aba Losi, Wolfgang Gärtner, Chen Song

**Affiliations:** 1 State Key Laboratory of Agricultural Microbiology, Huazhong Agricultural University, Wuhan 430070, China; 2 Institut für Analytische Chemie, Universität Leipzig, Linnéstraße 3, 04103 Leipzig, Germany; 3 Pflanzenphysiologie, Justus-Liebig-Universität, Senckenbergstraße 3, 35390 Gießen, Germany; 4 Department of Mathematical, Physical and Computer Sciences, University of Parma, 43121 Parma, Italy

**Keywords:** photoreceptor, cyanobacteriochrome, tongue region, chromophore-binding pocket, solid-state NMR, site-directed mutagenesis, structural modeling

## Abstract

Unlike canonical phytochromes, the GAF domain of cyanobacteriochromes (CBCRs) can bind bilins autonomously and is sufficient for functional photocycles. Despite the astonishing spectral diversity of CBCRs, the GAF1 domain of the three-GAF-domain photoreceptor all2699 from the cyanobacterium *Nostoc* 7120 is the only CBCR-GAF known that converts from a red-absorbing (Pr) dark state to a far-red-absorbing (Pfr) photoproduct, analogous to the more conservative phytochromes. Here we report a solid-state NMR spectroscopic study of all2699g1 in its Pr state. Conclusive NMR evidence unveils a particular stereochemical heterogeneity at the tetrahedral C3^1^ atom, whereas the crystal structure shows exclusively the *R*-stereochemistry at this chiral center. Additional NMR experiments were performed on a construct comprising the GAF1 and GAF2 domains of all2699, showing a greater precision in the chromophore–protein interactions in the GAF1-2 construct. A 3D Pr structural model of the all2699g1-2 construct predicts a tongue-like region extending from the GAF2 domain (akin to canonical phytochromes) in the direction of the chromophore, shielding it from the solvent. In addition, this stabilizing element allows exclusively the *R*-stereochemistry for the chromophore-protein linkage. Site-directed mutagenesis performed on three conserved motifs in the hairpin-like tip confirms the interaction of the tongue region with the GAF1-bound chromophore.

## 1. Introduction

Phytochromes constitute a superfamily of photosensory proteins in which plant phytochromes are the best-known members [1,2]. Members were identified in higher and lower plants, in fungi, in photosynthetic bacteria—in which cyanobacteria sport the greatest wealth of phytochromes—and also in non-photosynthetic bacteria. A joint feature of phytochromes is the employment of bilins as chromophores that are bound autocatalytically to the protein via conserved cysteine residues, and also the domain architecture of their photosensory module (PSM) comprising three domains, namely PAS (*P*eriod/*A*rnt/*S*ingleminded), GAF (c*G*MP-phosphodiesterase/*a*denylate cyclase/*F*hlA), and PHY (*phy*tochrome-specific). Phytochromes exhibiting this tridomain arrangement are considered to be canonical phytochromes. Despite the common use of bilins as chromophores, there are variations about the location of chromophore attachment: Phytochromes from fungi and those from bacteria (except cyanobacteria) bind to a cysteine located in the N-terminal extension (NTE), whereas all others carry the instrumental cysteine in the GAF domain. In fact, the variation of the chromophore-binding site is paralleled by a variation of the chromophore: Bacteriophytochromes employ biliverdin IX*α* (BV) whereas phytochromes from plants and most in cyanobacteria carry phytochromobilin (PΦB) or phycocyanobilin (PCB) [2]. Interestingly, irrespective of the location of the instrumental cysteine, the chromophore is embedded in the GAF domain in similar conformations. Photochemical switching between the 15*Z* dark state and 15*E* photoproduct is probably also common to all phytochromes [3,4,5,6]. Intriguingly however, in the ‘bathy’ subgroup of bacteriophytochromes the dark state is *E*, and the photoproduct is *Z* [7,8,9]. Double-bond photoisomerization is accompanied by a strong shift of the S_0_ → S_1_ bilin absorption maxima in the dark and photoproduct states (photochromicity) with a wavelength difference of >100 nm in some cases [2].

**Figure 1:**
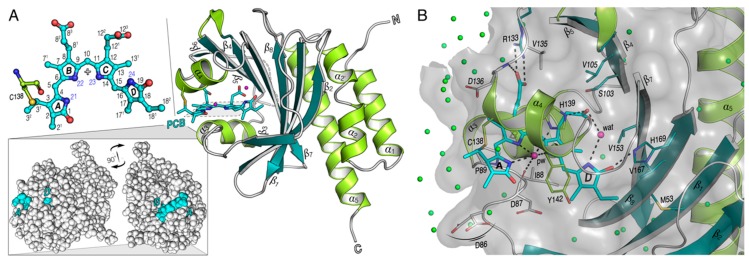
X-ray crystallographic structure of all2699g1 from *Nostoc* 7120 (PDB code 6OZA). (**A**) Ribbon diagram of the Pr form of all2699g1. The prominent helices (light green) and sheets (dark green) of the main chain are labeled following the Cph1 convention [23]. The central helices *α*_3_ and *α*_4_ and the anti-parallel sheets *β*_1_–*β*_8_ form the chromophore-binding pocket. The PCB chromophore (cyan) and its thioether linkage to C138 of the protein (light green) are shown as sticks. The disordered loop region (H118–E128) is indicated as a dotted gray line. Insets on the left side (top), the PCB and its covalent attachment to C138. The PCB chromophore within its binding site adopts a C5-*Z*,*syn*/C10-*Z*,*syn*/C15-*Z*,*anti* (*ZZZssa*) geometry. Pyrrole rings ***A***–***D*** and PCB atom numbers are indicated. (bottom) Space-filling model showing the exposed chromophore (cyan) in the GAF1 domain of all2699. (**B**) Structural view of the chromophore pocket. Key hydrogen-bond contacts between the chromophore and its binding pocket are indicated by dotted black lines, water molecules in the tetrapyrrole cavity as pink spheres. The phytochrome-like pyrrole water (pw) is present on the *α* face of the chromophore, see also Figure S5. Another strategically-placed water (wat) connecting the ***D***-ring pyrrole nitrogen (N24) to the ***C***-ring propionate carboxylate group is shown explicitly. The chromophore-binding pocket is open to the hydrating waters (green spheres).

A recent survey in cyanobacteria revealed a novel subgroup of phytochrome-like proteins with remarkable, entirely unexpected properties [10,11]. These cyanobacteriochromes (CBCRs) share with phytochromes the capability to ligate bilin chromophores autocatalytically by the formation of a thioether linkage between the conserved cysteine of the protein and the ethylidene group on the C3 side chain of the bilin ring ***A*** (Figure 1A). In contrast to canonical phytochromes, however, CBCRs are composed of GAF domains in repetitive arrangements. Up to seven GAF domains can be present, as seen in PtxD of *Nostoc punctiforme* [12], but also single-GAF CBCRs have been described [11,13]. CBCRs with multiple GAF domains can have one or more that bind PCB [14,15,16,17]. In fact, even single CBCR-GAF domains (or GAF domains individually expressed from an array of such domains) can bind the PCB chromophore and undergo photoconversion [6,14,17,18]. CBCRs can utilize PCB as a chromophore, similar to other canonical phytochromes such as Cph1 from the cyanobacterium *Synechocystis* 6803 and plant phytochromes [19,20], yet they differ with respect to their domain architectures, to their capability to modify the chromophore chemically, and to their absorption maxima.

Several outstanding features make CBCR-GAF domains interesting research objects: Their small size, high photochemical stability, large extinction coefficient and fluorescence quantum yields up to 15%. Moreover, they are able to modulate the activities of various enzyme modules to which they have been fused translationally, rendering them promising tools for optogenetic applications [21,22]. The second significant feature of CBCRs compared to canonical phytochromes is their wide range of absorption maxima of dark and photoproduct states. Plant phytochromes carrying phytochromobilin (PΦB) as chromophore absorb near 665 nm in the red-absorbing Pr dark state and 730 nm in the far-red-absorbing Pfr photoproduct, whereas their cyanobacterial orthologs like Cph1 carrying PCB as chromophore absorb at 650 and 718 nm, respectively. CBCRs, in contrast, span virtually the entire visible spectrum and even show absorption in the near ultraviolet (UV/A) region [11,15].

A large subgroup of CBCRs are formed as a red-absorbing dark state at *λ*_max_ ~ 650 nm that is photoconverted into a green-absorbing photoproduct at *λ*_max_ ~ 530–540 nm [1,6,17,18]. However, so far only one CBCR-GAF domain has been described that—from its red-absorbing dark state—undergoes a *batho*chromic shift of its absorption maximum upon irradiation. The gene *all2699* from the cyanobacterium *Nostoc* 7120 encodes a protein with three consecutive GAF domains and carries in its C-terminal part a canonical histidine kinase [14]: all2699g1 and all2699g3 each bind a PCB chromophore with dark state absorption maxima at *λ*_max_ ~ 638 and 645 nm, respectively. Whereas all2699g3 shows a *hypso*chromic shift of its *λ*_max_ to ~ 595 nm (orange-absorbing), *λ*_max_ of the all2699g1 photoproduct is shifted *batho*chromically to ~ 685 nm. Thus, this stand-alone GAF1 domain with only 199 amino acids might serve both as a useful model system for the much larger, more complicated canonical phytochromes with PAS–GAF–PHY architecture, as well as a potential optogenetic regulatory unit. Intriguingly, the GAF2 domain of all2699 shows sequence and structural similarity to the PHY domain of canonical phytochromes.

We, therefore, carried out a comprehensive solid-state cross-polarization magic-angle spinning (CP-MAS) NMR study of the Pr states of two all2699 constructs: (*i*) GAF1-domain-only (all2699g1) and (*ii*) GAF1-GAF2 (all2699g1-2), both carrying a uniformly ^13^C- and ^15^N-labeled PCB chromophore. The GAF1-2 construct was generated to test whether a potential interaction between the two GAF domains exist, analogous to the situation in phytochromes where the PHY domain carries a tongue-like extension that protrudes towards the GAF domain, interacting with and stabilizing the bilin chromophore [5,7,9,23,24]. The tongue in the Pr dark state comprises an antiparallel *β*-sheet structure that upon irradiation is converted into a short *α*-helix [4,25,26,27]. The stabilizing role of the tongue was further probed by site-directed mutations of amino acids comprising three highly conserved tongue motifs. For these NMR investigations on all2699g1 and all2699g1-2 proteins, we used the PSM of the canonical cyanobacterial phytochrome Cph1 (Cph1Δ2) as a reference for the NMR analysis [28,29,30,31,32,33] (see alignment of the sequences in Figure S1). NMR data provided information on the chromophore geometry, flexibility of the GAF1-bound chromophore and its interactions with nearby amino acids. This is compared in constructs with and without the GAF2 domain and also with the 3.0 Å crystal structure of all2699g1 (Figure 1, PDB code 6OZA, made available by personal communication from Yang, Xiaojing, UIC) and the structural model of the all2699g1-2 conduct.

## 2. Results

### 2.1. NMR Spectroscopic Analyses

#### 2.1.1. ^13^C Chemical Shift Assignments of the Chromophore in the Two all2699 Constructs

**Figure 2:**
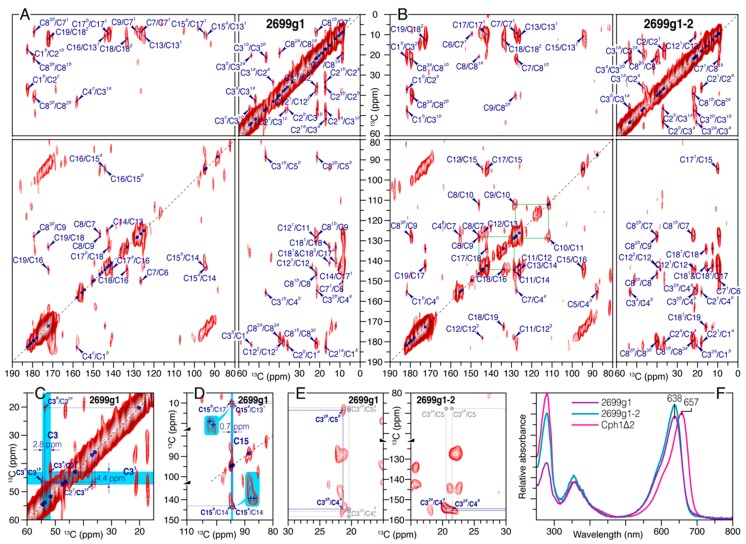
The PCB chromophore is structurally heterogeneous in the all2699g1 and all2699g1-2 dark states. 2D ^13^C–^13^C DARR spectra of the *u*-[^13^C,^15^N]-PCB chromophore in all2699g1 (**A**) and all2699g1-2 (**B**) with a mixing time of 50 ms. Both direct ^13^C–^13^C connectivities and indirectly-bonded carbon pairs of the PCB chromophore (see Figure 1A for numbering) are indicated by arrows and labeled in blue and their corresponding off-diagonal counterparts marked + in blue (see also Figures S2 and S3 for enlarged contour plots of the two DARR spectra with 1D traces projected on both dimensions). Observed ^13^C signal doublings and triplings of a subset of carbon resonances are superscripted with *a*, *b*, and *c*, respectively, from the high to the low field side. Correlation network splits for the ***A***-ring carbons and the C15 methine bridge of all2699g1 illustrated in (**C**) and (**D**), respectively (expanded views are *inset*). (**E**) Non-bonded correlations involving C3^2^ such as C3^2^/C4 and C3^2^/C5 allowing determination of the stereochemistry at the C3^1^ atom in all2699g1 and all2699g1-2, see also Figure 3E. Unresolved ^13^C pairs are indicated by open circles and labeled in gray. (**F**) Dark-state UV-vis absorbance spectra of all2699g1, all2699g1-2, and Cph1Δ2 following irradiation at 730 nm.

We previously reported the PCB chromophore chemical shifts for canonical phytochromes in both photostates typified by the cyanobacterial Cph1 that here serves as a reference protein [28]. Following a similar strategy, we obtained complete ^1^H, ^13^C and ^15^N assignments for the same bilin in the respective Pr dark states of all2699g1 and all2699g1-2. We started with acquisition of ^13^C–^13^C DARR spectra of both proteins with a long mixing time of 50 ms (Figure 2A,B for all2699g1 and all2699g1-2, respectively, enlarged views with 1D traces on both dimensions in Figures S2 and S3) which was found to be most efficient in recording medium- and long-range carbon atom pairs like C7^1^/C9 and C7^1^/C8^3^ (Figure 1A for numbering) of the PCB chromophore within its pocket. In all2699g1, the C10-methine carbon shows no correlations with neighbors but was identified in the 1D ^13^C MAS spectrum (see Figure S2). This unresolved carbon is assigned unambiguously in all2699g1-2, however, by using a well-defined correlation network connecting C8–C9–C10–C11–C12 (traced by green solid lines, Figure 2B). Indeed, the all2699g1-2 bilin shows more one-bond and indirect correlation pairs than all2699g1, particularly for those involving pyrrolic carbons. This is most likely due to the fact that DARR is a spin-diffusion based NMR approach, and its efficiency is associated with the size and rigidity of a molecule [34]. In any case, a number of long-range correlations like C9/C7^1^, C18^1^/C17, and C16/C13^1^ are fully resolved in all2699g1 (Figure 2A). This set of carbon pairs, according to the crystal structure (PDB code 6OZA) have internuclear C···C distances of 3.3–3.7 Å, roughly the effective range at this mixing time for the detection of long-range transfers. On the other hand, a similar detection limit of 3.3–3.6 Å is defined in all2699g1-2 (Figure 2B).

Both DARR spectra allowed unambiguous assignment of the propionate side chains of rings ***B*** and ***C***. The latter formed by C12^1^, C12^2^, and C12^3^ showed only a single correlation network analogous to that observed in the Cph1Δ2 dark state [29], whereas for the ***B***-ring propionate (C8^1^, C8^2^, and C8^3^) at least two sets of chemical shifts are visible in both all2699 bilins. The all2699g1 data also revealed a signal tripling for its carboxylate moiety (C8^3^) with a ^13^C resonance separation of 2.3 ppm (Table S1). Besides the ***B***-ring propionate, there are many such ^13^C signal splittings observed for both all2699 bilins, pointing to a conformational heterogeneity of the PCB chromophore in these proteins. Considering all2699g1-2, only a subset of ***A***-ring carbons shows splitting of correlation networks, whereas in all2699g1, all ***A***-ring carbons and even the C5-methine carbon bridging rings ***A*** and ***B*** are involved (Table S1). Furthermore, again in all2699g1, a 0.7-ppm resonance separation is observed for C15-methine (Figure 2D) and a 1.2-ppm separation for C17 (Table S1) in ring ***D***. The doublings in the ***D***-ring region observed specifically for all2699g1 are intriguing, since no such splittings are apparent with presence of the GAF2 domain in all2699g1-2 (Table S1).

**Figure 3:**
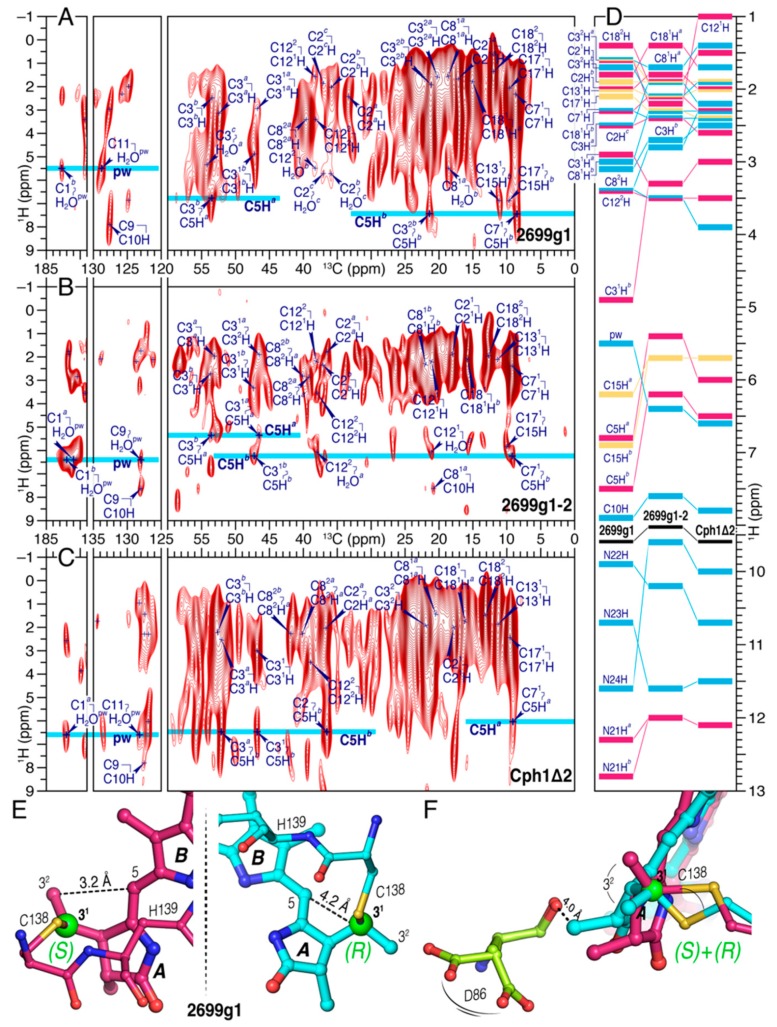
PCB chromophore positioned in a more flexible binding pocket in dark-state all2699g1 relative to the situations in all2699g1-2 and Cph1Δ2. 2D ^1^H–^13^C supercycled PMLG-decoupled dipolar correlation spectra of the *u*-[^13^C,^15^N]-PCB chromophore in all2699g1 (**A**), all2699g1-2 (**B**), and Cph1Δ2 (**C**) with a contact time of 1 ms at a cut-off distance of ~3.8 Å [30]. For all three dark-state spectra, the characteristic spectral region of *δ*^H^ = −1–9 ppm (*ω*_1_-dimension) displayed for tracing mostly direct ^1^H–^13^C connectivities of the chromophore (indicated by arrows and labeled in blue, see also Figure S4 for the full contour plots of (**A**)–(**C**) with the complete assignments of intramolecular ^1^H^N21–N24^–^13^C^PCB^ and interfacial ^1^H^residue/water^–^13^C^PCB^ correlations). For a given bilin proton such as C5H showing multiple chemical shifts, the signals are designated as C5H*^a^* and C5H*^b^*. ^1^H sites of pw, C5H*^a^*, and C5H*^b^* are highlighted in cyan. C5H correlations to the ethylidene side-chain carbons such as C3^1^ and C3^2^ served to corroborate the previously assigned stereochemistry at the C3^1^ atom. (**D**) ^1^H chemical shifts of the all2699g1 chromophore in comparison to those of all2699g1-2 and Cph1Δ2, see also Tables S2 and S3. Each ^1^H resonance is represented by a solid rectangle and labeled in blue. Multiple resonances from a given proton are colored the same. The spectral region without resonances (*δ*^H^ = 8–9.5 ppm) is omitted from the figure. (**E**) Chiral center at the C3^1^ position (shown as green sphere) of the all2699g1 chromophore found to occur in both *S*- and *R*-configurations. The *R*-stereochemistry at C3^1^ atom corresponds to the all2699g1 6OZA structure (Figure 1), whereas a model of *S*-stereochemistry at this position was built according to the AnPixJg2 3W2Z structure [35]. Key stereochemical restraints are indicated by dashed lines and the corresponding interatomic distances are labeled in black. (**F**) A stereochemical mixture at the tetrahedral C3^1^ atom. D86 on the *β* face of the chromophore (near ring ***A***) is shown as two alternative side-chain rotamers implying a heterogeneous microenvironment of the chromophore (see also Figure 1B).

In all2699g1, the most pronounced ^13^C signal splittings occur at the ***A***-ring region in terms of the number of split components of the carbon resonances and magnitude of their separations. Specifically, we identified a double set of chemical shifts for the carbonyl (C1), a methyl substituent (C3^2^), and C5. Moreover, the signals from other ***A***-ring carbons C2, C3, C3^1^, and C4 are evidently tripled, with a resonance separation of 3.5, 2.8, 4.4, and 3.9 ppm, respectively. These separations are much larger than those observed in the canonical Cph1 phytochrome (Table S1), and thus, cannot be merely associated with a local decrease in the degree of order or multiple marginally-heterogeneous protein surroundings. In addition to the large ^13^C resonance separations found at the ***A***-ring covalent linkage to the protein such as C3 and C3^1^ in all2699g1 (Figure 2C), C3^2^ of the ethylidene side chain resolved two correlations with the C4 and C5 carbons in the ***A***–***B*** methine bridge (Figure 2E). Considering a detection limit of 3.3–3.7 Å with our current sensitivity, this set of correlations is critical in judging the chirality at the ethylidene C3^1^ position (Figure 3E). The internuclear C···C distance between C3^2^ and C5 in the *R*-stereoisomer is 4.2 Å (extracted from the all2699g1 crystal structure), thereby clearly outside the detection range. For the *S*-enantiomer, instead, the intramolecular transfer from C3^2^ to C5 would be over a distance of ~2.9–3.2 Å, well within the detection range, and thus, should be seen. The distance estimation was extracted from the equivalent carbon pairs in the crystal and solution NMR structures of red/green CBCRs AnPixJg2 and NpR6102g4, both showing a clear *S*-configuration at the C3^1^ atom [6,35,36]. Such a situation is indeed seen in Figure 2E for all2699g1, the correlations of C3^2*b*^ with C4*^a^* and C5*^b^* are fully visible, and, on the other side, no such correlations appear for C3^2*a*^ with C4 and C5, thus, supporting the possible coexistence of *S*- and *R*-stereoisomers at the ***A***-ring C3^1^ position (Figure 3F). In all2699g1-2 (and to note also in the crystal structure of GAF1), however, the chiral center at C3^1^ is exclusively in the *R*-configuration (within the detection limits) since both C3^2^ species correlate only with C4 (at a distance of 3.7 Å) but not with C5 (Figure 2E).

#### 2.1.2. ^1^H–^13^C Correlation Spectra for Cph1Δ2 and the Two all2699 Protein Samples

The stereochemistry at the C3^1^ atom of the two all2699 bilins is further strengthened by the ***A***-ring ^1^H–^13^C correlations of the chromophore (Figure 3). In both all2699 constructs and Cph1Δ2, the C5-methine proton (C5H) adapts two different ^1^H sites (Figure 3A–C) which are separated by 0.5–0.8 ppm (Figure 3D and Table S2). The HETCOR spectra reveal a number of correlations between the ***A***-ring ethylidene carbons (like C3 and C3^1^) and C5H, but only a single one resolved for C3^2^ in all2699g1 (C3^2*b*^/C5H*^b^*, Figure 3A). This correlation signal can solely be identified in the *S*-stereoisomer by considering the selectivity of the HETCOR experiment in terms of the cut-off distance for heteronuclear transfers. By choosing a contact time of 2 ms, a cut-off distance of ~3.8 Å is defined, corresponding to a heteronuclear dipolar coupling of around 200 Hz [37]. This is not overestimated as shown by the fully-resolved all2699g1 correlation involving C11 of ring ***C*** and the pyrrole water resonating at *δ*^H^ of 5.5 ppm (Figure 3A). In contrast to C3^2*b*^ in the *S*-stereoisomer, C3^2*a*^ in the *R*-stereoisomer is too far removed from C5H (>4 Å) for magnetization transfer. This assignment is further validated by the absence of C3^2^/C5H correlations in both all2699g1-2 and Cph1Δ2, indicating that the C3^1^ atom of ring ***A*** is not stereochemically heterogeneous but occurs exclusively in the *R*-configuration. In addition, two C3^1^H sites (C3^1^H*^a^* and C3^1^H*^b^*) are seen specifically for all2699g1 at 2.9 and 4.9 ppm, respectively (Figure 3A and Table S2). The former has a similar ^1^H shift to the C3^1^H protons in all2699g1-2 and Cph1Δ2 (resonating at 3.3 and 3.0 ppm, respectively, Figure 3D), it is associated with the observed *R*-stereoisomer. The latter, C3^1^H*^b^*, is associated with the *S*-stereoisomer and shows a 2.0-ppm downfield shift. This could arise, for example, due to rotation of the thioether linkage (Figure 3E,F). The observed ^1^H separation at C3^1^H is consistent with a larger separation at C3^1^ (4.4 ppm), the signal of which is tripled (Figure 2A and Table S1). Besides C2H, the all2699g1 HETCOR data revealed at least two sets of ^1^H chemical shifts for other ***A***-ring protons. Amongst these, a split of the correlation network of C4 and the proton bound to the ***A***-ring pyrrole nitrogen, N21H, is identified (C4*^a^*/NH21*^a^* and C4*^b^*/NH21*^b^*, Figure S4), however, this small but rather intense doubling is retained in neither all2699g1-2 nor Cph1Δ2 (Figure 3D and Figure S4). Similarly, a 0.7-ppm ^1^H resonance separation is observed for the proton of C5-methine carbon bridging rings ***A*** and ***B*** (Figure 3D).

Part of ^1^H correlations in the spectra shown in Figure 3A–C is not associated with bilin protons but attributed to the interfacial transfer of polarization from protons of water molecules. For example in all2699g1, three ^1^H sites at around 5.4, 5.5, and 5.7 ppm were recognized (Figure 3A). Considering the potential interfacial contacts of C1 and C11 within the detection range of ~3.8 Å, the ^1^H site centered at around 5.5 ppm most likely originates from the centrally-positioned pyrrole water. The latter is hydrogen-bonded to the pyrrole nitrogens of rings ***A***–***C***, and in addition, bridges D87 of the DIP motif to the conserved H139 (homologous to D207 and H260 in Cph1) on the opposite side of the bilin (Figure 1B and Figure S5). The ^1^H signal of this water molecule shifts to 6.4 ppm in the presence of the GAF2 domain (Figure 3B) and further to 6.9 ppm in Cph1 (Figure 3C), indicative of a more deshielded environment for the water protons. This effect could arise due to either a modification of relative spatial disposition of the pyrrole water to electronegative elements nearby (such as the pyrrole nitrogens and imidazole nitrogen of H139) or a redistribution of delocalized positive charge of the bilin. Further prominent ^1^H changes associated with small distortions of hydrogen-bonding geometry were also detected for the two NH protons of rings ***B*** and ***C***, both are 0.8 ppm downfield shifted in Cph1 relative to those in all2699g1 (Figure 3D and Table S3).

#### 2.1.3. ^15^N Assignments of Four Pyrrole Nitrogens in the Two all2699 Proteins

We next performed 1D ^15^N CP/MAS experiments on Cph1Δ2 and all2699 proteins (Figure S6). The two all2699 spectra manifest a relatively stronger protein backbone signal expected from natural abundance than Cph1Δ2 which arises from a less efficient bilin incorporation in the all2699 proteins (based on the determination of extinction coefficients [14]), paralleling the situations in red/green and violet/orange CBCRs represented by AnPixJg2 [32] and NpF2164g3 [38], respectively. The low holoprotein concentration impeded an unequivocal assignment of the ^15^N signals of the two all2699 through direct ^13^C–^15^N correlations, so instead the all2699 ^15^N assignments were based on our recent DNP-enhanced MAS NMR work on Cph1Δ2 [31]. The characteristic spectral region of 125–170 ppm for the signals of the four pyrrole nitrogens in both all2699 spectra display far-reaching similarities with Cph1Δ2, and thus, the relative order of the four ^15^N signals in Cph1Δ2 is the main argument for tentative all2699 assignments (Figure S6 and Table S4). The narrow dispersion of ^15^N shifts (around 30 ppm) observed in both all2699 spectra (Figure S6A,B), similar to that in Cph1Δ2 (Figure S6C), indicates that all four nitrogens are fully protonated, and thus, the bilin is positively charged (ignoring the propionate side chains) [31,32]. Taking the four ^15^N signals in Cph1Δ2 as a reference, both bilin nitrogens of the two inner rings ***B*** and ***C***, N22 and N23 are shifted upfield by 2.1 and 1.4 ppm, respectively in all2699g1-2, and a further upfield shift of 0.9 ppm is observed for N23 in all2699g1. Moreover, in all2699, all four bilin nitrogens appear to be weakly affected by the presence of the GAF2 domain in terms of ^15^N chemical shift, in which the largest ^15^N shift of 1.0 ppm occurs at N24. Interestingly, this change is linked to a significant upfield ^1^H shift of its directly-bound proton N24H from 11.6 to 9.6 ppm, indicative of a modification of hydrogen-bonding interaction with a structural water (Figure 1B). However, all four ^15^N signals are sharper in all2699g1-2 than in all2699g1 (Table S5). Major line-narrowing is seen for ***A***-ring N21 and ***C***-ring N23.

#### 2.1.4. Comparison of ^1^H, ^13^C, and ^15^N Chemical Shifts of the Bilin in all2699g1 with all2699g1-2 and Cph1Δ2

**Figure 4:**
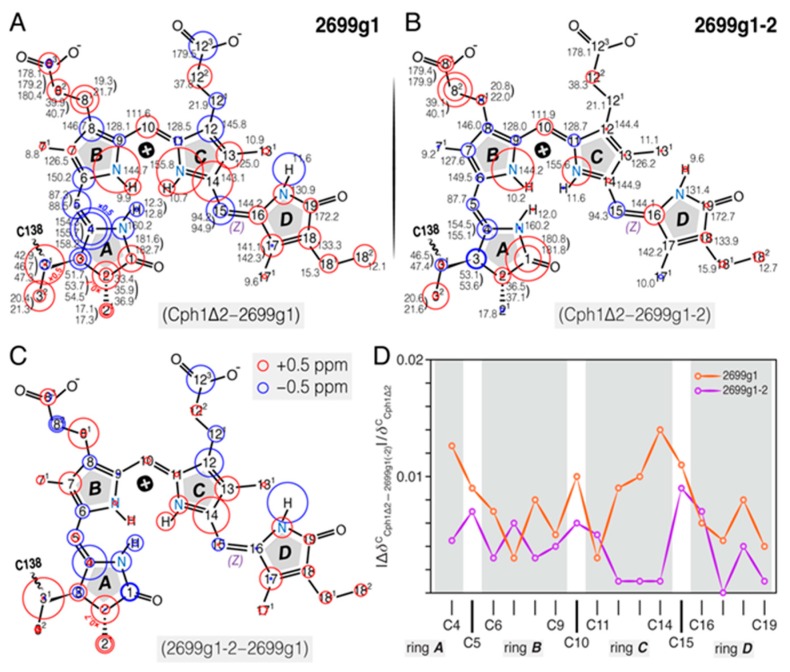
Comparison of electronic structure and immediate environment of the chromophore in all2699g1 and all2699g1-2 with Cph1Δ2 in their dark states. The ^13^C, ^15^N (N21–N24), and ^1^H (H^N21–N24^) chemical shifts of the dark-state chromophore in all2699g1 (**A**) and all2699g1-2 (**B**) are labeled in black numbers and the corresponding changes to those of Cph1Δ2 are represented as red and blue circles for down- and up-field shifts, respectively, see also Tables S1, S3 and S4 . Atoms showing signal splitting are labeled with multiple circles. (**C**) Impact of the GAF2 domain in all2699 protein on the chemical shifts of the GAF1-bound chromophore. Red and blue circles represent down- and up-field shifts, respectively. (**D**) Relative changes in ^13^C chemical shifts of the carbon atoms in the *π* chain of all2699g1 (orange) and all2699g1-2 (purple) to Cph1Δ2. Changes for each *π*-conjugated carbon (shown as spheres) were calculated as |Δ*δ*^C^_Cph1Δ2 – 2699g1(-2)_|/*δ*^C^_Cph1Δ2_.

The global differences of ^1^H, ^13^C, and ^15^N chemical shifts of the incorporated PCB bilin for all2699 (g1 and g1-2) and Cph1Δ2 in their respective dark states (Figure 4) yield remarkable information on the role of the GAF2 domain in all2699. A number of important features of all2699g1 bilin in comparison with Cph1 were found: (*i*) More numerous ^1^H and ^13^C signal splittings, particularly noticeable for the ***A***-ring region, (*ii*) nearly all the ^13^C signals of rings ***A*** and ***D*** move downfield (red circles in Figure 4A), amongst which the major changes occur at C3^1^ and C3^2^. Moreover, most of the other carbons exhibit moderate shifts ranging from 0.4 to 1.1 ppm (Table S1), (*iii*) the C4–C6 atoms associated with the ***A***–***B*** methine bridge undergo a collective upfield shift of 4.1, 1.4, and 1.1 ppm, respectively, and (*iv*) larger ^15^N deviations are localized at N22 and N23 of the two inner rings, whereas N21 and N24 of the outer rings are less affected. Figure 4B depicts the chemical shifts of the all2699g1-2 bilin and the differences with respect to Cph1Δ2. Comparing Figure 4A,B, a global decrease in difference magnitude was seen in the latter case, with almost all carbons of rings ***C*** and ***D*** showing only subtle changes (<0.5 ppm). The main exceptions are C1 of the ***A***-ring carbonyl and C8^2^ of the ***B***-ring propionate. Furthermore, the chemical shift changes caused by the presence of the GAF2 domain in all2699g1-2 (Figure 4C) are characterized by (*i*) a nearly identical up- and down-shift pattern with smaller magnitudes relative to Figure 4A, in particular for atoms in the ***B***–***D*** regions except for C8^2^ and C16, (*ii*) the signs of the changes are also reversed for some carbons from the hydrophobic side of the ***A***-ring region (such as C3^1^, C4, and C5), and (*iii*) unlike the large ^15^N shifts revealed for N22 and N23 in Figure 4A, none of the pyrrole nitrogens exhibit a comparable chemical shift change, for example, N22 moves only 0.9 ppm, whereas N23 is essentially unaffected (Table S4). Moreover, in all2699g1-2, the ^13^C shifts of the *π*-conjugated C4–C19 system are quantitatively more similar to those in Cph1 than all2699g1 (Figure 4D).

### 2.2. Characterization of all2699g1-2 proteins

In vivo and in vitro PCB chromophore assembly yield different absorption maxima in some recombinant CBCRs. Notably, slr1393g3 from *Synechocystis* 6803 exhibits red/green photochemistry in its native form, whereas in vitro assembly yields an orange- (*λ*_max_ ~ 587 nm) instead of the green-absorbing photoproduct [18]. As for the NMR investigation in this study, isotopically-labeled PCB chromophore with apoprotein at natural abundance was required, chromophore assembly was examined for all2699g1 and all2699g1-2 following both in-vivo and in-vitro assembly protocols. Differences between the absorbance maxima of neither dark state nor photoproduct in these proteins were detected (data not shown). Moreover, as can be seen in Figure 2F, the absorbance maximum of the all2699g1-2 Pr dark state is identical to that of all2699g1 (*λ*_max_ ~ 638 nm), whereas its Pfr photoproduct showed a 20-nm red shift (*λ*_max_ ~ 705 nm) relative to that of all2699g1 (*λ*_max_ ~ 685 nm), indicating a strong interaction between the GAF2 domain and the GAF1-bound chromophore especially in the Pfr state (see below).

**Figure 5:**
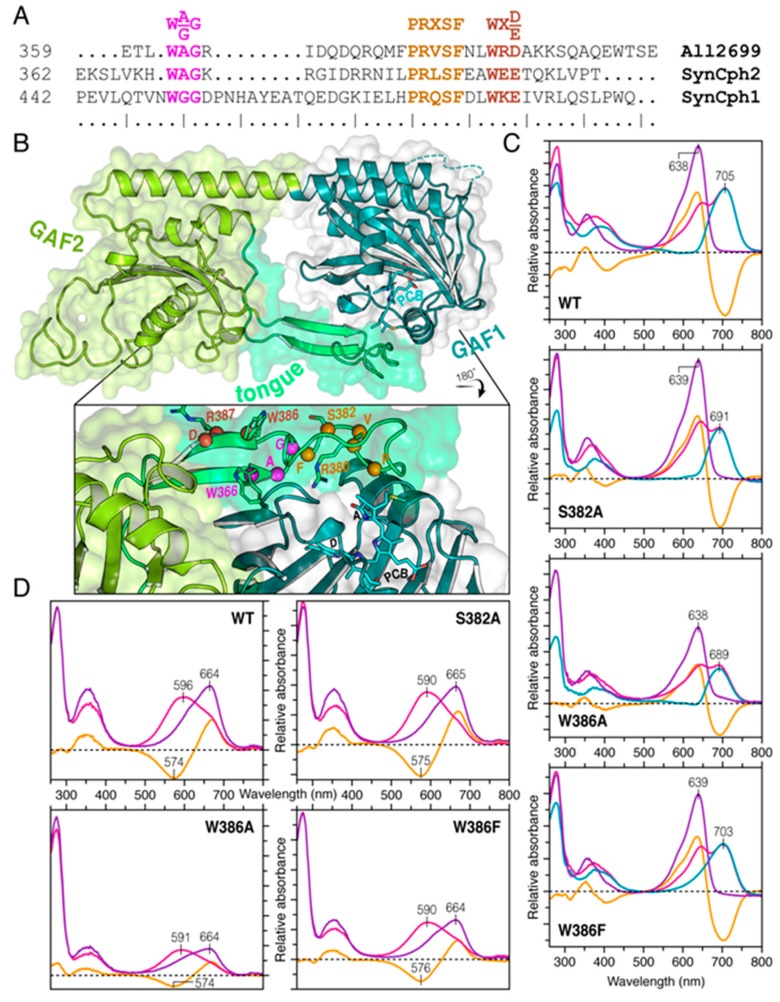
A tongue-like protrusion from the GAF2 domain shields the GAF1-bound chromophore from the solvent. (**A**) Conserved residues in the tongue region of all2699 from *Nostoc* 7120 (UniProtKB/Swiss-Prot entry Q8YTL8), cyanobacterial Cph2 (Q55434), and Cph1 (Q55168). The three conserved W(A/G)G, PR*X*SF, and W*X*(D/E) motifs are labeled and colored purple, yellow, and brown, respectively. (**B**) A structural homology model of all2699g1-2 constructed based on the crystal structures of the GAF1 domain (PDB code 6OZA) and Cph2(1-2) module (PDB code 4BWI). The PCB chromophore (cyan) in the GAF1 domain (dark green) is shielded from the solvent by the tongue (light green) protruding from the GAF2 domain (yellowish green). *Inset*, a detailed view of the tongue/GAF1 interface with their molecular surfaces colored light-green and gray, respectively. Conserved residues within the tongue and those used to construct the variants of this protein are shown as spheres and sticks, respectively, and labeled in the appropriate color as in (**A**). (**C**) UV-vis absorbance spectra of wild type all2699g1-2 module and three representative variant proteins containing single substitutions at the tongue residues within the conserved motifs (S382A, W286A, and W386F). Spectra are shown for the dark state (Pr, purple) and after saturating red irradiation (Pr/Pfr photoequilibrium mixture, red) with absorption maxima indicated. Difference spectra calculated as Pr minus Pr/Pfr photoequilibrium and deconvoluted Pfr spectra are shown in orange and blue, respectively. (**D**) Absorption (Pr, purple, and Pfr, red) and absorption difference (orange) spectra of wild type and three variant proteins after denaturation with acidic urea. See also Figure S8 for analysis of additional four all2699g1-2 variant proteins containing substitutions for W366, R380, and R387 within the conserved tongue motifs.

The crystal structure of all2699g1-2 in its Pr state shows an overall structural similarity with that of a Cph2-type phytochrome (Figure S7) [25]. The structural model reveals a tongue-like extension protruding from its PHY-like GAF2 domain (Figure 5B) which interacts with the PCB-binding pocket of the GAF1 domain via three highly-conserved motifs in an almost invariable manner. The stabilizing role of the tongue has been studied already in Cph2, revealing the conserved tongue motifs that upon mutation strongly alter the spectral and kinetic properties of the molecule [39]. The three conserved motifs are W(G/A)G, PR*X*SF, and W*X*(D/E) (*X* = variable, amino acid positions in all2699g1-2 being W366, P379, and W386, respectively, Figure 5A,B). Sequence comparison between all2699g1-2 and Cph2 reveals strong conservation in these motifs (Figure 5A). Accordingly, in all2699g1-2 both Trp residues were mutated to Ala or Phe (W366A/F and W386A/F, Figure 5C and Figure S8A,B). Additionally, S382 was converted to Ala (S382A, Figure 5C) and also R380 and R387 as potentially structurally relevant amino acids were converted (R380I and R387P, Figure S8C,D). For all variants, the Pr form remains unchanged or show only marginal shifts whereas in some cases the absorption maxima of Pfr forms undergo remarkable shifts upon mutation (W386F, *λ*_max_ ~ 703 nm; S382A, 691 nm; R387I, 704 nm, summarized in Table S6). Still, despite changes in the Pfr absorptions maxima, the photochemistry of the mutated variants remains intact, as can be seen from the absorbance spectra of the proteins after denaturation (Figure 5D and Table S6).

## 3. Discussion

### 3.1. Bilin Chromophore in the Pr dark States

We collected the complete ^1^H, ^13^C, and ^15^N chemical shifts of the PCB chromophore embedded in all2699g1 and all2699g1-2. The ^1^H chemical shifts of the PCB protons in Cph1Δ2 were also determined (Figure 3D and Figure 4). A comparison of the ^13^C chemical shifts of the pyrrolic carbons of rings ***B*** and ***C*** in all three proteins reveals much similarity with a largest deviation of 2.0 ppm (C14, Table S1), indicating both coplanarity and rigidity of the two inner rings, as well as that the immediate protein interactions are similar in their respective Pr dark states. The 2.0-ppm deviation of C14 in all2699g1 relative to Cph1 might arise from a larger tilt angle between rings ***C*** and ***D*** in the former. The Cph1 2VEA structure indicates a shallow tilt of 27° [23], whereas that for all2699g1 (PDB code 6OZA) implies a 60° ***D***-ring rotation relative to the ***B***/***C***-ring plane. According to the Hückel theory, a tilted ***D***-ring geometry of >40° would impede conjugation across the C15-methine bridge, thus, leading to a blue shift in the absorbance *λ*_max_. This is indeed the case for the all2699g1 PCB in its dark state where *λ*_max_ is 19 nm blue shifted (at 638 nm) relative to that of Cph1 (at 657 nm, Figure 2F) whose ring ***D*** seems to be conjugated with the rest of the *π*-electron system. Pr crystal structures for Cph1 [23] and PΦB/BV-binding phytochromes [9,24,40] show a highly-conserved tyrosine triad which attends ring ***D***. Crystal structures imply that the position of the tyrosine side chain above the chromophore (263 in Cph1) determines the ***D***-ring tilt. The all2699g1 ^1^H interfacial contacts of the Y263-homologous residue to the PCB carbons are different from those in all2699g1-2 (Figure S4). For example, Hε2 of Y142 in all2699g1 is seen by the ***D***-ring carbons like C17 and C18^1^, implying proximity to the ***D***-ring methyl group. Instead, in all2699g1-2, the Hε2 proton interacts with C16, indicating a positional shift of the phenolic side chain further away from the methyl. The structural rearrangement of this Tyr would alleviate the ***D***-ring steric clash with its hydroxyl group, and thus, allow a more coplanar bilin conformation. In the presence of the GAF2 domain, the deviation of the C14 chemical shift value relative to Cph1 drops to 0.2 ppm (from 2.0 ppm as all2699g1) and a nearly uniform reduction is seen for pyrrolic carbons, particularly so for those associated with the ***C***–***D*** methine bridge and ring ***D*** (Figure 4D). The marginal ^13^C shift differences suggest similar *π*-orbital connectivity around ring ***D*** in both species. In this case, a shallower ***D***-ring tilt analogous to Cph1 would be expected.

The plausible ***D***-ring rotation associated with the GAF2 domain is also supported by a prominent 2.0-ppm ^1^H shift observed at the ***D***-ring N24H (Figure 3D). Analogous to the Cph1 2VEA structure, this pyrrole nitrogen and its ***D***-ring neighbor, C19 (carbonyl) are also involved in stabilizing the ring in its Pr position through hydrogen-bond interactions with a nearby structural water and the imidazole *ε*-nitrogen of H169 (290 in Cph1), respectively (Figure 1B). The GAF2 domain in all2699 is closely related to the PHY/GAF2 domains of canonical phytochromes like Cph1 [23] and their Cph2 homologs [25,39] that show a tight interaction between the GAF1 domain and the conserved tongue protrusion (see below). Mutagenesis and NMR studies suggest that the absence of the PHY domain might result in a stronger twist of the bilin ring ***D*** [41,42]. In fact, available CBCR crystal [17,18,35] and NMR-based [8] 15*Z* Pr structures support this notion. It is also important to note that the Pr absorption in all2699g1 is hardly altered by the presence of the GAF2 domain (Figure 2F). This indicates that the ***D***-ring out-of-plane rotation is not necessarily correlated with the absorption in the Pr dark state, consistent with a recent wave function analysis of slr1393g3 by Schapiro and coworkers [43].

C4, the other terminus of the *π*-conjugated chain, exhibits three ^13^C resonances in all2699g1 with an unusually large separation of 3.9 ppm (Table S1). In addition, a separation corresponding to 1.4 ppm is seen for the C5-resonances. These two carbons are associated with the ***A***–***B*** methine bridge and their ^13^C shifts are sensitive to slight geometric differences of the ***A***-ring conformers which could be derived from occurrence of both stereo-configurations for the C3^1^–Cys (C138) thioether linkage (see below). For instance, the Cph1 2VEA structure reveals a moderate tilt of 21° between rings ***A*** and ***B***, in which the stereocenter at the C3^1^ atom occurs exclusively in the *R*-configuration [23]. In contrast, the red/green-type CBCR AnPixJg2 3W2Z structure indicates that C3^1^ is *S*-configurated and its ring ***A*** is more coplanar with the ***B***/***C***-ring plane at an angle of 12° [35].

### 3.2. Heterogeneous Microenvironment of the GAF1-Bound PCB Chromophore in all2699 Proteins

Like C4 and C5, nearly all other ^1^H and ^13^C resonances in the all2699g1 ***A***-ring region are split up into a doubling or more (Figure 3D and Figure 4A), indicative of structural heterogeneity of the bilin in its Pr dark state. Such signal splitting is not confined to this ring but also seen in several regions of the chromophore, such as C15 associated with the ***C***–***D*** methine bridge, the ***B***-ring propionate (C8^1^, C8^2^, and C8^3^) and also C17 and C18^1^H of ring ***D***. This observation further supports the notion that both native phytochromes and CBCRs in their Pr dark states are heterogeneous population mixtures that can be distinguished structurally [4,6,18,35,44,45,46,47], biologically [48,49], spectrally [50,51,52,53,54,55] and photochemically [56,57,58,59,60,61]. The apparent ***A***-ring signal splitting is inherently linked to the heterogeneous attachment of the chromophore to C138 and the variable packing of ring ***A***. The ^1^H and ^13^C resonance separations observed for the ***A***-ring N21H and C1 carbonyl strongly imply structural plasticity of the local environment because of their direct involvements in an intricate hydrogen-bonding web within the pocket, e.g., N21 interacting with the pyrrole water and D87 from the invariant DIP motif at distances of 2.6 and 3.2 Å, respectively (Figure 1B). The heterogeneous ***A***-ring microenvironment in all2699g1 is further shown by the different side-chain conformations adopted by D86 (Figure 1B) which is adjacent to ring ***A*** with its main chain oxygen pointing towards C3^2^ (4.0 Å in the *R*-stereoisomer, Figure 3F). Moreover, the solution NMR structure for the Pr state of the red/green CBCR NpR6102g4 showed that the side chains of the Trp and Asp residues hydrogen-bonded to ring ***A*** adopt two alternative rotameric structures [6].

The flexibility and compatibility of the subpocket around ring ***D*** is also seen in all2699g1, and is even more evident in the Pr state of AnPixJg2. The reversible interconversion between the two Pr isomers showing different ***D***-ring geometry is accompanied by rearrangement of the hydrogen-bonding networks around the chromophore and modification in the solvation pattern of the binding pocket [62]. However, no NMR spectral heterogeneity is observed for ring ***D*** in all2699g1-2 and Cph1 (Table S1). A possible origin of this is increased rigidity around ring ***D*** which is shielded from the solvent by the tongue extending from the GAF2/PHY domains (Figure 5B). The tongue also partially covers the chromophore along rings ***A*** and ***B***, thus, enforcing order onto the carbons in the ***A***-ring region, as evidenced by weaker signal splitting in all2699g1-2 than all2699g1. The shielding effect is also manifested by broader ^15^N signals of the all2699g1 pyrrole nitrogens which are particularly evident for N21 and N23 of rings ***A*** and ***C*** (Table S5). Compared to the tripartite binding pocket for chromophore in Cph1, however, the two-GAF-domain pocket in the case of all2699g1-2 (Figure 5B) is not completely sealed off from the solvent due to deletion of the NTE region, perhaps promoting chromophore heterogeneity. The NTE region is known to be important for Pfr stability [47,63]. Moreover, a recent MAS NMR study of plant phytochrome also showed that the NTE adopts a more compact position closer to ring ***A*** in Pfr than in Pr [4].

### 3.3. Stereoselectivity of all2699 Apoproteins for PCB Chromophore

Besides the above-mentioned types of heterogeneity known in various CBCRs, all2699g1 shows stereochemical heterogeneity at the tetrahedral C3^1^ atom. This can be directly inferred from the homo- and heteronuclear correlations involving its ethylidene side-chain neighbor, C3^2^ with C5 and C5H (Figure 2E and Figure 3A). The ratio of the two stereogenic components would mainly rely on the covalent-binding reaction arising from a nucleophilic attack of a Cys thiolate anion to the C3^1^ position, in which the surrounding protein matrix plays a key role. The chromophore-binding pocket of all2699g1, in particular, the ***A***-ring microenvironment, is heterogeneous (Figure 1A). Indeed, available crystal structures of isolated CBCR-GAF domains [3,18,35] including all2699g1 (PDB code 6OZA) show a fairly water-exposed chromophore. This might lead to greater conformational variability of the chromophore due to missing GAF domains, the tongue of which would otherwise shield the chromophore from the outer medium. The C3 side chain would, thus, possess considerable conformational flexibility to favor the attack of the conserved Cys (Figure 3E). The non-stereoselectivity of this attachment, however, is not a general feature for the single CBCR-GAF domains such as the red/green CBCRs of slr1393g3, AnPixJg2, and NpR6102g4, all of which exhibit a Pr state similar to that of all2699g1. The stereochemistry at C3^1^ in the former case has been determined to be exclusively in *R* [18], opposite to the *S* stereochemistry of the two latter cases [6,35], probably because of the different steric requirements of the two stereoisomers. For the *S* stereochemistry at this position, twisting of the thiol side chain in the reactive Cys residue is always seen which would further cause a large displacement of this Cys and adjacent His (Figure 3E). Both residues are perfectly conserved amongst all known CBCRs and canonical phytochromes [1] and, moreover, the His → Leu mutation obliterates covalent attachment in plant phytochrome A [64]. In all2699, the homologous H139 is bridged via the pyrrole water to D87 from the DIP motif on the opposite side of the chromophore (Figure 1B). The stereochemical difference of the thioether bond certainly has an impact on the electrostatic environment, the position of the pyrrole water coordinated by H139, D87, and the three pyrrole nitrogens of rings ***A***–***C*** (Figure 1B and see below).

The stereochemical heterogeneity observed in all2699g1 is not retained in the presence of the GAF2 domain, but an *R* stereochemistry at C3^1^ is chosen by analogy to canonical cyanobacterial and Cph2-like phytochromes [23,25]. In this respect, it is interesting to note that the *R* stereochemistry is also exclusive in the Pr crystal form of all2699g1 (PDB code 6OZA). One may inspect crystal structures for hints on heterogeneity, however. Protein molecules in the crystal state usually adopt identical or similar conformations close to a local thermodynamic minimum, heterogeneous mixtures like C3^1^ stereoisomers being eliminated from the crystallizing ensemble. Retention of the *R*-stereochemistry in all2699g1-2 could arise from a transition to a more hydrophobic environment for the amphipathic ring ***A*** created by the presence of the GAF2 domain which would also provide space to accommodate a less strained ***A***-ring geometry. The stereoselectivity associated with the GAF2 domain in all2699 is consistent with the decreased flexibility of the chromophore in a more ordered binding pocket (see above).

### 3.4. The Pyrrole Water

2D ^1^H–^13^C HETCOR experiments on all2699 (g1 and g1-2) and Cph1 identified interfacial contacts between the PCB carbons and several structural water molecules including the pyrrole water (Figure 3A–C and Figure S4). This water molecule is essential for the formation of the hydrogen-bonding network connecting the ***A***, ***B***, ***C***-ring nitrogen triad to the binding pocket in all known canonical and Cph2-like phytochrome structures [23,24,25]. Although an analogous hydrogen-bonding lattice exists in all cases, the precise geometry is not identical, perhaps being fine-tuned by shifts of the pyrrole water in the cavity. As shown in the spectra for all2699g1 and Cph1 (Figure 3A,C), the pyrrole water correlates with ring ***A*** at C1. The relative intensity of the two correlations estimated from their integrated peak volumes in all2699g1 and Cph1 is 35:65, implying stronger heteronuclear transfer in Cph1 derived from closer disposition of the pyrrole water to C1, and thus, N21. Moreover, the relative positional shifts associated with the GAF2 domain are also clearly seen in the carbon contacts of the pyrrole water. For example, the contact with C11 of ring ***C*** is missing but with C9 as the N–C bond partner of the ***B***-ring N22 is fully resolved (Figure 3A,B). Indeed, compared to the situation in all2699g1, the pyrrole water in the Cph1 2VEA crystal structure is shifted closer to N21 and N22 of rings ***A*** and ***B*** (0.6 and 0.7 Å, respectively) but more distant from N*δ*1 of H139 (0.5 Å). A similar adjustment is also seen in the Cph2 4BWI structure (Figure S5). It should be also noted that the significant ^15^N shift differences observed between all2699g1 and Cph1 for N22 and N23 of the inner rings (Figure 4B) would imply that the positive charge held by the protonated bilin is distributed differently. The local electronic environment for the pyrrole water protons may, thus, be less shielded. These results indicate that the ^1^H shift of the pyrrole water in all2699g1 is not retained in all2699g1-2 and Cph1, but rather has moved downfield dramatically by ~1.0 ppm (Figure 3D). Moreover, intriguingly, this water molecule is clearly missing in available structures of the red/green CBCRs [6,18,35], contrary to all2699 and canonical phytochromes that show a red-shifted photoproduct absorption. It, therefore, appears that photoproduct tuning in CBCRs and phytochromes is related to the presence of pyrrole water.

### 3.5. The Tongue

The structural similarity between the PHY domain in canonical phytochromes to the GAF domain family and in particular the long, tongue-like extension it carries was first revealed in Cph1 [23]. The tongue stretches back to make contact with the adjacent GAF domain, thereby sealing the chromophore in its pocket. 3D crystal structures and sequence comparisons indicate that the tongue is a characteristic feature of phytochromes. Interestingly, photoactivation is associated with a radical refolding of the tongue, the two anti-parallel sheets in Pr being replaced by a helix in Pfr. Sequence comparison and mutation analyses indicate that all2699g2 carries a tongue with similar functionalities (Figure 5C and Figure S8) to Cph2 [39]. For example, W386A blocks the formation of Pfr (*λ*_max_ ~ 689 nm vs. 705 nm for the wild type protein), whereas W386F has little effect (*λ*_max_ ~ 703 nm compared to 705 nm for the wild type protein). S382A identifies a second significant interaction between protein and chromophore leading to a *λ*_max_ ~ 691 nm for the photoproduct vs. 705 nm for the wild type (note equivalent value for all2699g1 is 685 nm). Moreover, R380 from the PR*X*SF motif forms a salt bridge to the acidic D87 of the GAF1 domain, thus, connects the Pr chromophore to the tongue. A similar pattern was also seen in the earlier phytochrome structures [9,23,25]. In all2699g1, the absence of salt bridge R380 would mean that D87 is not held in place and might lead to greater mobility of the chromophore in the GAF1 pocket.

## 4. Materials and Methods

### 4.1. Sample Preparation

#### 4.1.1. Cloning and Expression

DNA encoding all2699g1 (aa 1–199) and all2699g1-2 (aa 1–430) were PCR-amplified from a full-length *all2699* clone [14] using primers listed in Table S7 (Eurofins) and ligated via NdeI and XhoI into pET30. Site-directed mutagenesis was performed with primers listed in Table S7. Transformation into competent *E. coli* cells (BL21(DE3)) and subsequent induction with IPTG at 1 mM at 16 °C allowed generation of C-terminally-H_6_-tagged apoproteins.

#### 4.1.2. Protein Production for NMR

Preparation of *u*-[^13^C,^15^N]-PCB chromophore, Cph1Δ2 apo- and holoproteins was described elsewhere [30]. For all2699, the cell pellets were resuspended in ice-cold 50 mM Tris pH 8.0, 0.2 M NaCl buffer and disrupted ultrasonically (0 °C, pulse on: 1 s, pulse off: 2 s, total duration time of 24 min, Nanjing Safer Biotech). The lysate was then clarified by centrifugation at 15,000 rpm for 60 min at 4 °C and *u*-[^13^C,^15^N]-PCB (500 nmol) added to the supernatant at room temperature in darkness. Autoassembly was complete within 30 min. The samples were then purified via Ni^2+^-affinity chromatography on chelating Sepharose (GE Healthcare), holoproteins being eluted with 50 mM Tris pH 8.0, 0.5 M imidazole. Subsequently, imidazole was removed by dialysis against 50 mM Tris pH 8.0, 0.2 M NaCl, 3% *v/v* glycerol. The holoprotein solutions were concentrated using Amicon filters (ultra centrifugation filters, NMWCO 10 or 30 kDa for all2699g1, and all2699g1-2 and Cph1, respectively) to 100 μL at 4 °C by centrifugation at 4500 rpm. The holoproteins of 3.1, 5.1 and 6 mg of all2699g1, all2699g1-2, and Cph1Δ2, were loaded into a 1 mm bore glass capillary syringe and irradiated with 730 nm light for 30 s prior to loading into 4-mm ZrO_2_ MAS rotors under 730 nm LED (20 nm FWHM) light and snap-frozen in liquid N_2_ for subsequent NMR measurements.

#### 4.1.3. UV-vis Spectroscopic Analyses

Diluted aliquots of all samples were first investigated by UV-vis absorbance spectroscopy. Full photoconversions were ensured by irradiation with 590 and 730 nm light for 30 s from an array of appropriate LEDs. Reversibility of photochemistry was confirmed via 590 → 730 → 590 nm irradiation cycles. All the measurements were conducted at 20 °C.

### 4.2. NMR Spectroscopic Analyses

All MAS NMR data were acquired on a Bruker AVANCE-III 400 MHz WB NMR spectrometer (Rheinstetten, Germany) equipped with a 4-mm double-resonance MAS probe at −25 ± 0.2 °C maintained by a temperature control unit. The rotor was inserted into the precooled MAS stator and spun at 500 Hz upon freezing, ensuring homogeneous sample distribution throughout the rotor. The MAS rate of 8 kHz in ^13^C experiments and 3.5 kHz in ^15^N experiments was maintained ± 3 Hz with a Bruker MAS control unit. Optimized ^1^H, ^13^C, and ^15^N *π*/2 pulse lengths were 2.5, 4.1, and 4.7 µs, respectively. CP was optimized to satisfy *n* = ±1 Hartmann–Hahn conditions with ^1^H power ramped 70–100% [65] and a spin-lock field of 74.7 and 32.4 kHz for ^13^C and ^15^N, respectively. Swept-frequency two-pulse phase modulation heteronuclear decoupling [66] with 100 kHz r.f. strength was used during acquisition. ^13^C and ^15^N chemical shifts were referenced to the C(O)O^–^ signal of solid _L_-tyrosine·HCl at 172.1 ppm and the NH_4_^+^ signal of solid ^15^NH_4_NO_3_ at 23.5 ppm, respectively.

1D ^15^N CP spectra were acquired with a contact time of 2 ms and 122,880 scans with a relaxation delay time of 3.0 s. Line-broadening of 50 Hz was applied prior to Fourier transformation. 2D ^13^C DARR experiments were carried out with an optimized mixing time of 50 ms and a CP contact time of 2 ms. The *n* = +2 rotary-resonance condition was achieved with ^1^H continuous wave irradiation at r.f. field strength of 16 kHz. The spectra were acquired with 114 *t*_1_ increments, accumulating 1984 scans in each indirect slice with a relaxation delay time of 1.89 s. 2D ^1^H–^13^C HETCOR experiments were carried out with a CP contact time of 2 ms. Homonuclear ^1^H dipolar decoupling was achieved with supercycled phase-modulated Lee–Goldburg (PMLG5-S2) scheme [67,68]. Each PMLG5 block consists of 10 pulses with the following phases: 339.22°, 297.65°, 256.08°, 214.51°, 172.94°, 352.94°, 34.51°, 76.08°, 117.65°, and 159.22° (m5m shape in TopSpin 3.2 library, Bruker). A consecutive PMLG5 block was then repeated with 180° phase shift to complete the S2 supercycle. The optimized PMLG5 pulse was 1.33 µs with 88.5 kHz r.f. strength. Optimization was done by observing the *J*-splitting in adamantane powder by recoding PMLG5-S2-decoupled CP spectra, and further fine-optimized by monitoring the splitting between the CH_2_ protons of the solid *α*-glycine in the indirect dimension in ^1^H{PMLG5-S2}–^1^H{*w*PMLG5-S2} homonuclear correlation experiment [69]. The scaling factor of 0.32 was then calculated by dividing the observed difference between the centers of the CH_2_ and NH_3_ signals in the indirect dimension by their difference in a static sample of 4.84 ppm. Dividing the spectral width by the scaling factor appropriately scaled the ^1^H indirect dimension which was referenced by assigning the midpoint of two CH_2_ proton peaks of the *α*-glycine to 3.52 ppm. The 2D HETCOR spectra were acquired with 60 *t*_1_ increments, accumulating between 2048 and 3024 scans in each indirect slice with a relaxation delay time of 3.5 s. A 45° shifted squared sine-bell window function was applied in the indirect dimension, and further zero-filled to 1024 points prior to Fourier transformation. A 90° shifted squared sine-bell window function was applied in the direct dimension and zero-filled to 4096 data points.

### 4.3. Construction of Structural Model for all2699g1-2 Pr dark State

The structural homology model of all2699g1-2 (aa 11–406) was built in two steps using SWISS-MODEL and ClusPro with the aid of the DeepView tool (Swiss-PdbViewer, v4.1). In the first step, the crystal structure of the Cph2(1-2) photosensory module (aa 4–418, PDB code 4BWI) was selected as template. The sequence identity with respect to all2699g1-2 was 32%, the GMQE (global model quality estimation) fairly good at 0.65 [70]. In the second step, the GAF2 domain of the model (residues 186–406) was then docked to the crystal structure of all2699g1 (PDB code 6OZA) using the ClusPRO server for protein-protein docking and energy minimization [71]. The dimer ranked as most reliable by the ClusPro docking was selected also given the high similarity with Cph2 in the mutual orientation of the two GAF domains. The tongue region (strand-loop-strand) of all2699g2 comprises aa 361–391 with D373 and M377–P379 of the loop in close vicinity to the chromophore. In Cph2 the tongue encompasses aa 363–395 with R378–P382 of the loop in close vicinity to the chromophore.

## 5. Conclusions

In this work, we used MAS NMR to characterize the electronic structure, conformational flexibility and interplay of the PCB chromophore with the GAF1 domain of all2699, with and without the associated GAF2 domain. Besides a pronounced structural heterogeneity of the PCB chromophore in all2699g1, our data reveal the coexistence of *S*- and *R*-stereoisomers at the ***A***-ring C3^1^ chiral center. In the presence of GAF2, however, an exclusively *R* stereochemistry is seen, consistent with the decreased flexibility of the chromophore in all2699g1-2. The structural model of all2699g1-2 (Figure 5B) and mutagenesis data (Figure 5C,D and Figure S8) confirm that GAF2 makes close contact with the GAF1-bound chromophore via conserved motifs in the tongue. We consider that the more ordered chromophore pocket associated with the presence of GAF2 arises from shielding of the chromophore from the solvent and the newly-formed D87–R380 salt bridge connecting the chromophore to the tongue in Pr. Our data provide insight into the interactions between chromophores and their binding pockets in many photoreceptor families in particular canonical phytochromes.

## Supplementary Materials

Supplementary materials can be found at https://www.mdpi.com/1422-0067/20/15/3656/s1.
