# Supplementary material for: MAS NMR on a Red/Far-Red Photochromic Cyanobacteriochrome All2699 from Nostoc
Source: Int J Mol Sci. 2019 Jul 26;20(15):3656. doi: 10.3390/ijms20153656 (PMC6696110; doi:10.3390/ijms20153656)
Supplement: Supplementary file 1 [file ijms-20-03656-s001.pdf]

Supplementary Materials

MAS NMR on a red/far-red photochromic cyanobacteriochrome all2699 from *Nostoc*

Qian-Zhao Xu[1,2], Pavlo Bielytskyi[2], James Otis[2], Christina Lang[3], Jon Hughes[3], Kai-Hong Zhao[1,*], Aba Losi[4], Wolfgang Gärtner[2], and Chen Song[2,*]

[1]State Key Laboratory of Agricultural Microbiology, Huazhong Agricultural University, Wuhan 430070, China; [2]Institut für Analytische Chemie, Universität Leipzig, Linnéstraße 3, 04103 Leipzig, Germany; [3]Pflanzenphysiologie, Justus-Liebig-Universität, Senckenbergstraße 3, 35390 Gießen, Germany; [4]Department of Mathematical, Physical and Computer Sciences, University of Parma, Parco Area delle Scienze 7/A-43124 Parma, Italy.

[*]Correspondence: chen.song@uni-leipzig.de (C.S.); khzhao@163.com (K.-H.Z.).

This pdf file includes:

Figures S1–S8

Tables S1–S7

References

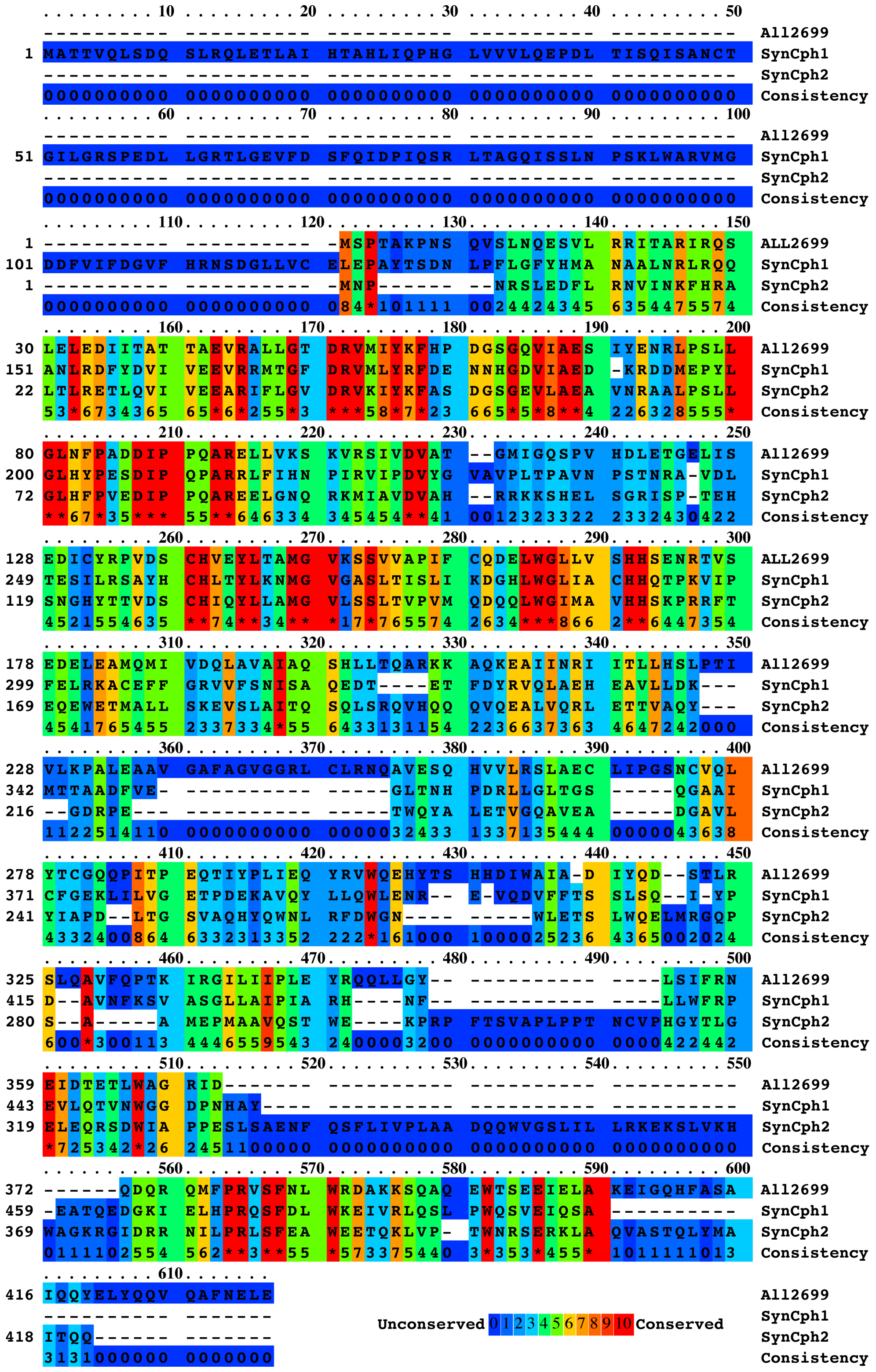

Figure S1. Sequence alignment of all2699g1-2 and cyanobacterial Cph1 and Cph2. The sequence alignment was performed by PRALINE [1]. The scoring scheme ranges from 1 for the least conserved alignment position to 10 for the most conserved alignment position (conserved amino acids indicted by asterisks).

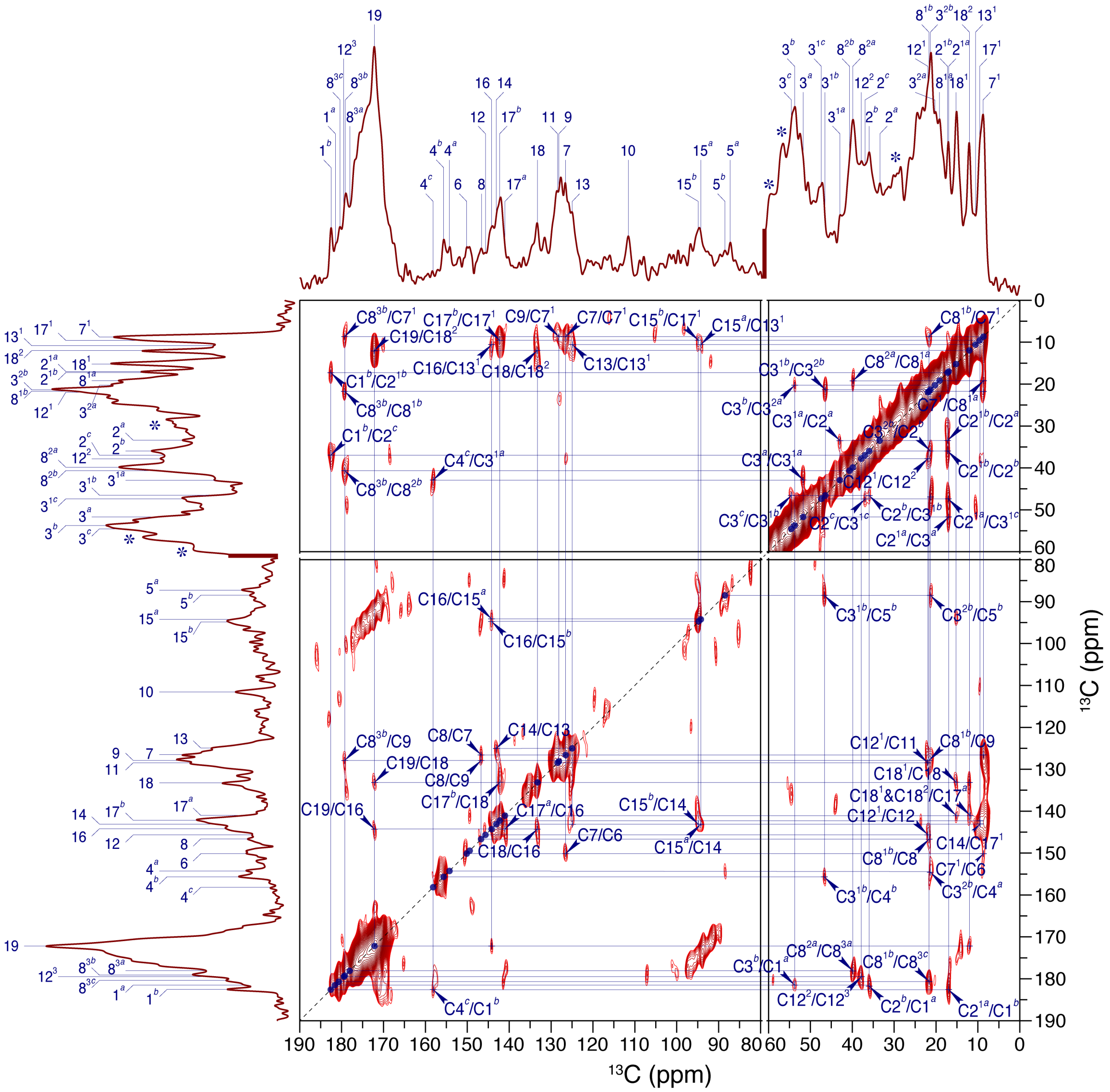

Figure S2. Enlarged contour plot of the DARR spectrum shown in Figure 2A. The DARR spectrum of *u*-[^{13}C,^{15}N]-PCB-all2699g1 as Pr was acquired with a mixing time of 50 ms. The 1D traces of the 2D spectrum (along ω_1-, *left*, and ω_2-dimension, *top*) are shown with the assignment of ^{13}C peaks (see Figure 1A for chromophore numbering). Asterisks indicate peaks arising from the natural abundance glycerol carbons. Correlation signals are marked '+' and labeled in black with their corresponding off-diagonal counterparts marked only.

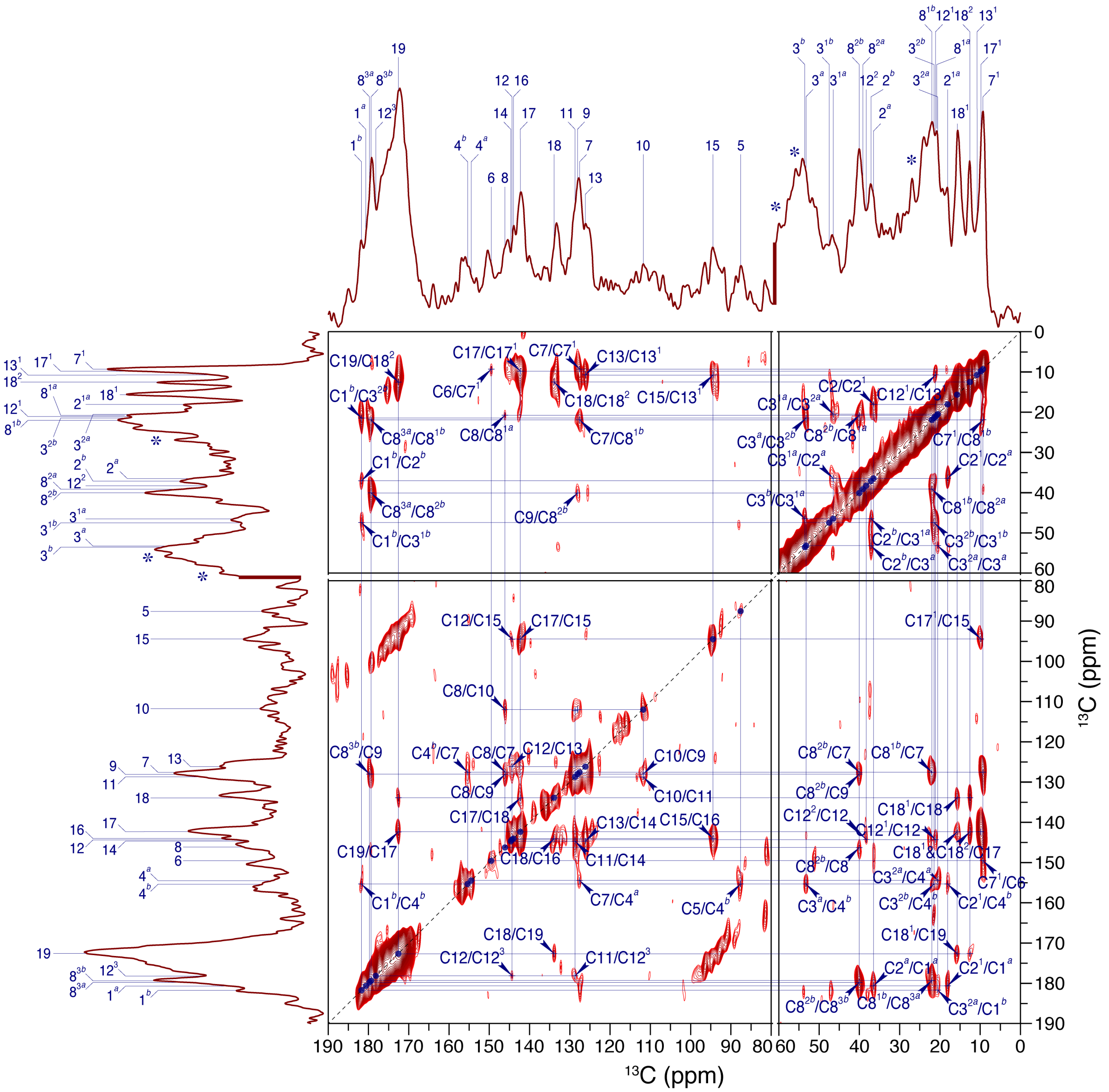

Figure S3. Enlarged contour plot of the DARR spectrum shown in Figure 2B. The DARR spectrum of *u*-[^{13}C,^{15}N]-PCB-all2699g1-2 as Pr was acquired with a mixing time of 50 ms. The 1D traces of the 2D spectrum (along ω_1-, *left*, and ω_2-dimension, *top*) are shown with the assignment of ^{13}C peaks (see Figure 1A for chromophore numbering). Asterisks indicate peaks arising from the natural abundance glycerol carbons. Correlation signals are marked '+' and labeled in black with their corresponding off-diagonal counterparts marked only.

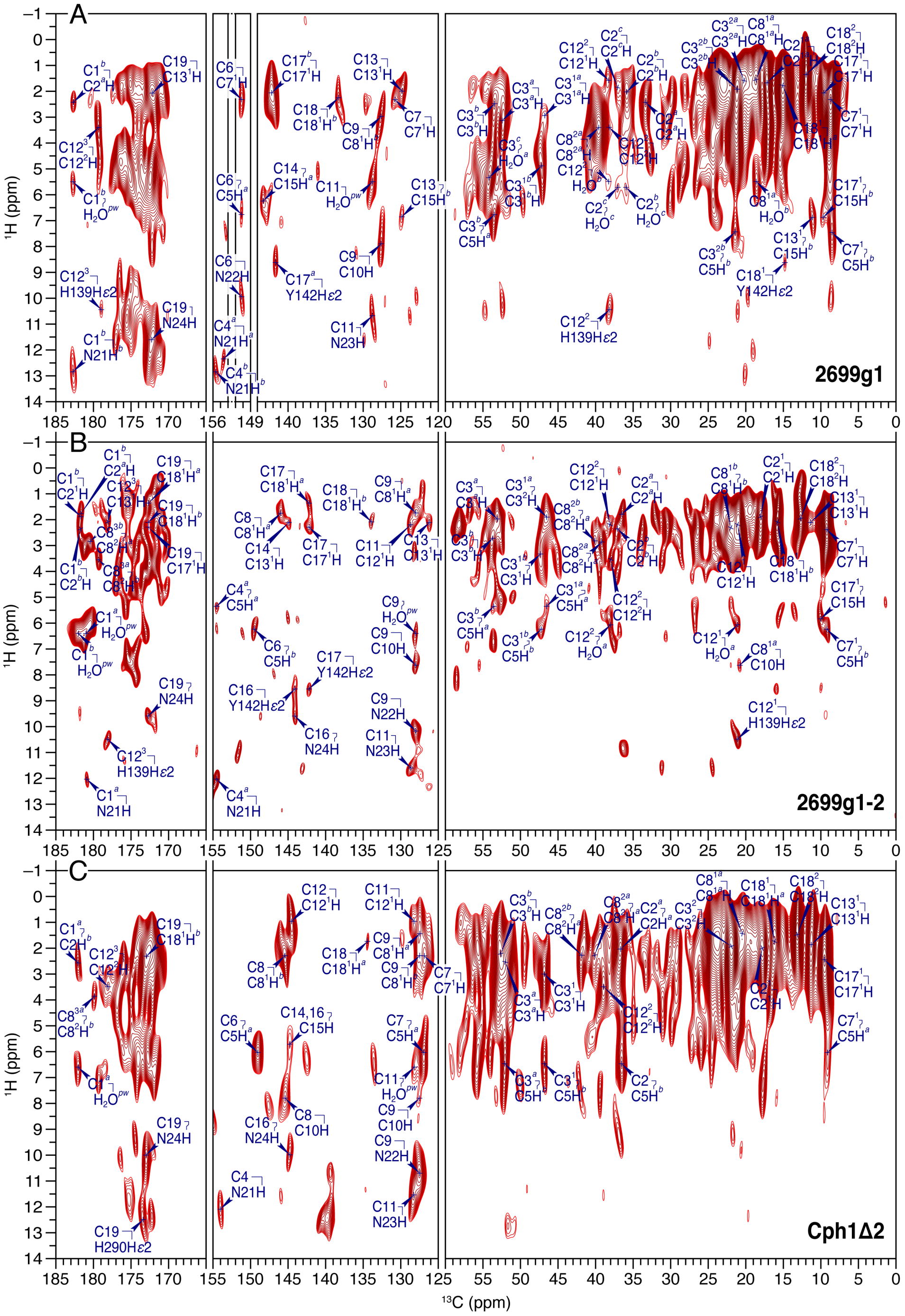

Figure S4. Full contour plots of the HETCOR spectra shown in Figure 3A–C. 2D ^1H–^{13}C supercycled PMLG-decoupled dipolar correlation spectra of the *u*-[^{13}C,^{15}N]-PCB chromophore in all2699g1 (**A**), all2699g1-2 (**B**), and Cph1Δ2 (**C**) with a contact time of 1 ms. The complete assignments including intramolecular $^1H^{N21–N24}$–$^{13}C^{PCB}$ and interfacial $^1H^{residue/water}$–$^{13}C^{PCB}$ correlations are shown. ^1H chemical shifts of the PCB protons in all the three photosensors are summarized in Tables S2 and S3 and illustrated in Figure 3D.

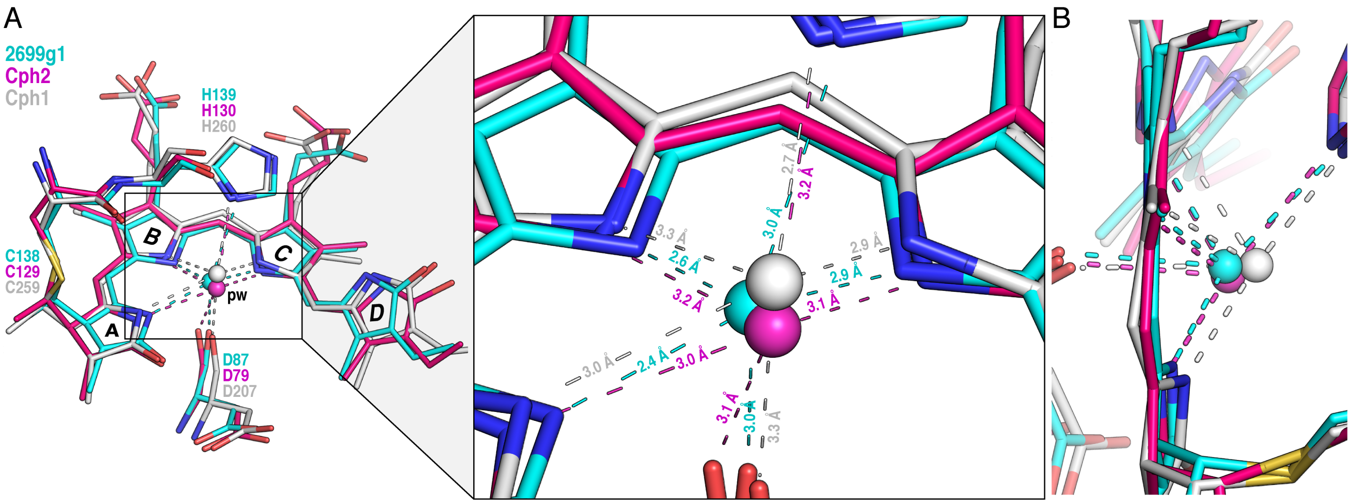

Figure S5. Disposition of the pyrrole water in the Pr-state binding pocket of all2699g1 with respect to that of Cph1 [2] and Cph2 [3]. (**A**) Interactions of the pyrrole water (shown as sphere) with the highly-conserved His/Asp residues and the ***A***–***C***-ring pyrrole nitrogens in all2699g1 (cyan), Cph1 (gray), and Cph2 (pink). (**B**) The subsites are displayed in side orientations.

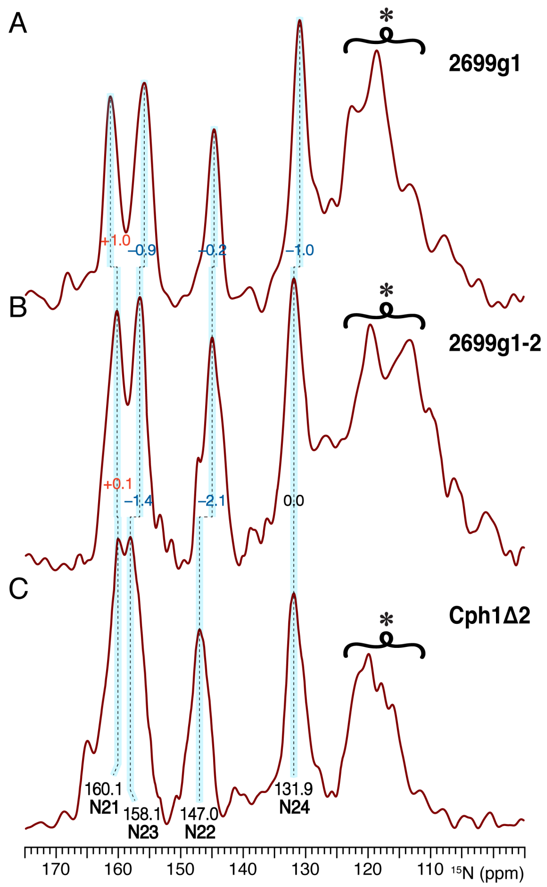

Figure S6. All four pyrrole nitrogens are protonated in the all2699g1 and all2699g1-2 Pr dark states yielding a positively charged tetrapyrrole system. ^{15}N CP/MAS NMR spectra of the *u*-[^{13}C,^{15}N]-PCB in all2699g1 (**A**), all2699g1-2 (**B**), and Cph1Δ2 (**C**) measured with a CP contact time of 2 ms. ^{15}N chemical shifts (δ^N) are indicated by the vertical dashed lines and peak assignments for the two all2699 samples based on conclusive ^{15}N assignments of Cph1Δ2 dark state (labeled at the bottom) by using DNP-enhanced MAS NMR [4]. The δ^N differences between Cph1Δ2 and all2699g1-2 as well as between all2699g1-2 and all2699g1 are traced and labeled in blue and red for up- and down-field shift, respectively (summarized in Table S4). Protein signals arising from backbone amide are denoted by asterisks.

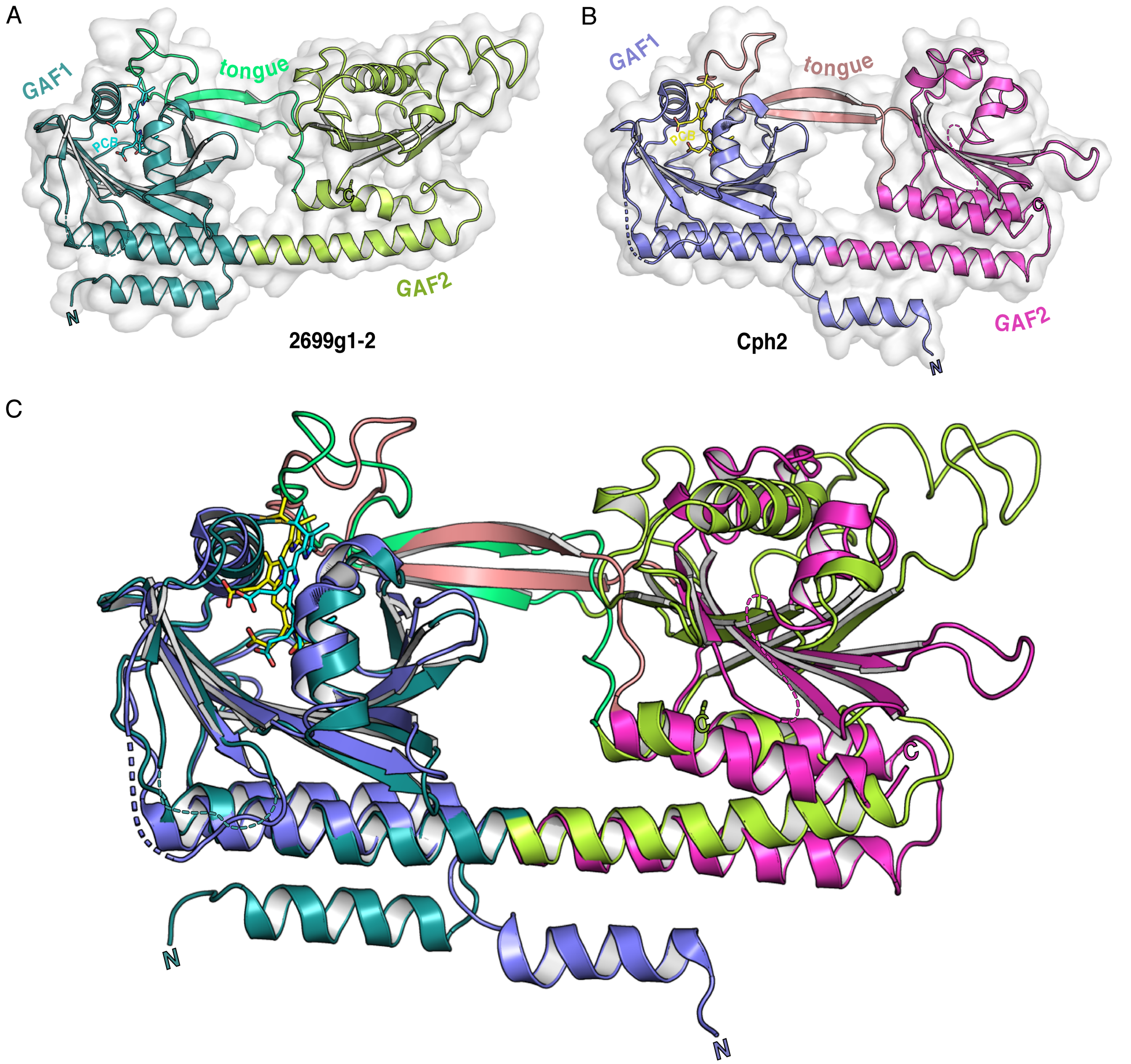

Figure S7. Structural model of all2699g1-2 construct in its Pr state and comparison to the crystal structure of Cph2 [3]. (**A**) Structural homology model of all2699g1-2 constructed based on the crystal structures of GAF1 domain and Cph2 module. The PCB(cyan)-bound GAF1, GAF2, and tongue protrusion from the GAF2 domain are depicted in cyan, yellow-green, and lawn-green, respectively. (**B**) Cph2(1-2) 4BWI Pr structure. The GAF1, GAF2, and tongue protrusion are depicted in blue, pink, and pale red, respectively. The PCB is in yellow. (**C**) Superimposition of all2699g1-2 structural model and the Cph2 4BWI structure.

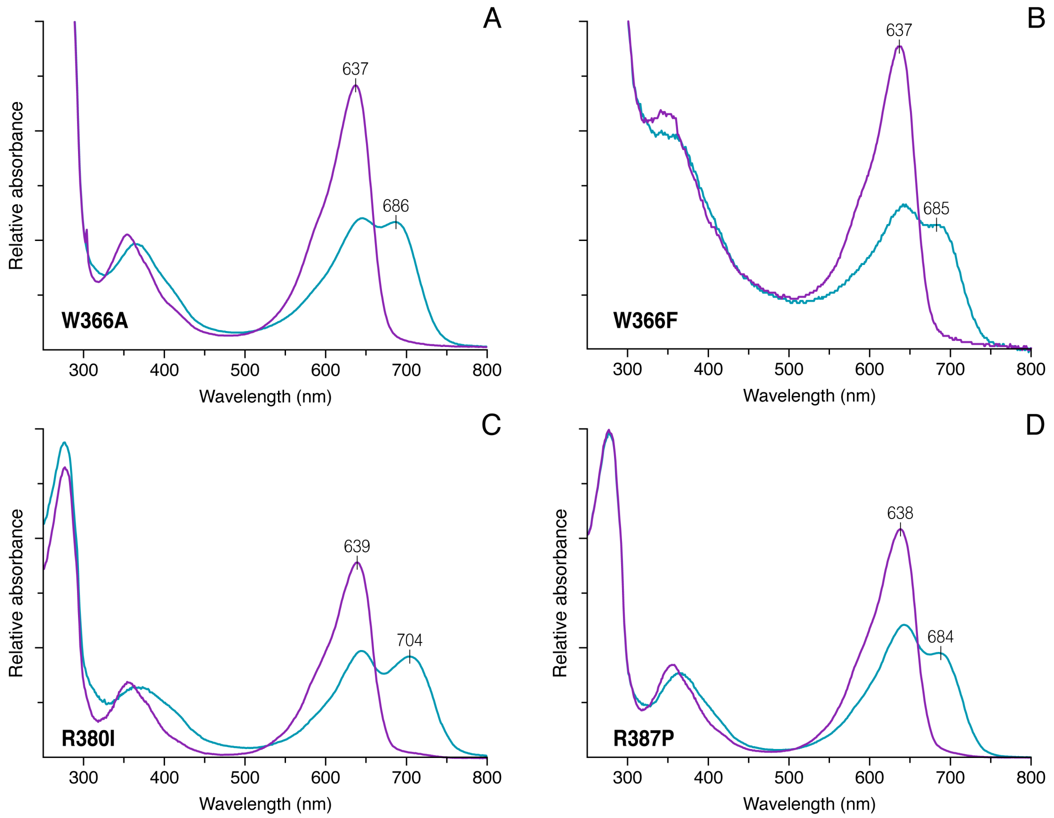

Figure S8. UV-vis absorbance spectra of tongue variants in all2699g1-2. The residues of the three conserved motifs in the tongue region are shown in Figure 5A. The spectra of W366A (A), W366F (B), R380I (C), and R387P (D) were measured after far-red (Pr state, purple) and red-light illumination (Pfr, green line). The corresponding spectra of the wild-type and three more variant proteins (S382A, W386A, and W286F) are shown in Figure 5C.

Table S1. Overview of ^{13}C chemical shifts of the PCB chromophore incorporated in all2699g1, all2699g1-2, and Cph1Δ2 in their respective Pr dark states. Published ^{13}C data of Cph1Δ2 [5] were listed for reference. The ^{13}C chemical shift differences of PCB chromophore in the three photoreceptors, $\Delta_{2699g1\text{-}2-2699g1}$, $\Delta_{Cph1\Delta2-2699g1}$, and $\Delta_{Cph1\Delta2-2699g1\text{-}2}$ are reported and illustrated in Figure 4. The chromophore numbering is according to Figure 1A.

PCB carbons		^{13}C chemical shift (ppm)			^{13}C chemical shift difference (ppm)		
		2699g1	2699g1-2	Cph1Δ2	$\Delta_{Cph1\Delta2}$ − 2699g1	$\Delta_{Cph1\Delta2}$ − 2699g1-2	$\Delta_{2699g1\text{-}2}$ − 2699g1
ring A	**1**	181.6 (1^a)	180.8 (1^a)	182.1 (1^a)	+0.5	+1.3	−0.8
		182.7 (1^b)	181.8 (1^b)	184.0 (1^b)	+1.3	+2.2	−0.9
	**2**	33.4 (2^a)	-	-	-	-	-
		35.9 (2^b)	36.5 (2^a)	36.7 (2^a)	+0.8	+0.2	+0.6
		36.9 (2^c)	37.1 (2^b)	37.9 (2^b)	+1.0	+0.8	+0.2
	**2^1**	17.1 (2^{1a})	18.0	17.8	+0.7	−0.2	+0.9
		17.3 (2^{1b})			+0.5		+0.7
	**3**	51.7 (3^a)	53.1 (3^a)	52.1 (3^a)	+0.4	−1.0	+1.4
		53.7 (3^b)	53.6 (3^b)	52.7 (3^b)	−1.0	−0.9	−0.1
		54.5 (3^c)	-	-	-	-	-
	**3^1**	42.9 (3^{1a})	-	46.8	+3.9	-	-
		46.7 (3^{1b})	46.5 (3^{1a})		+0.1	+0.3	−0.2
		47.3 (3^{1c})	47.4 (3^{1b})		−0.5	−0.6	+0.1
	**3^2**	20.4 (3^{2a})	20.6 (3^{2a})	21.9	+1.5	+1.3	+0.2
		21.3 (3^{2b})	21.6 (3^{2b})		+0.6	+0.3	+0.3
	**4**	154.3 (4^a)	154.5 (4^a)	154.1	−0.2	−0.4	+0.2
		155.7 (4^b)	155.1 (4^b)		−1.6	−1.0	−0.6
		158.2 (4^c)	-		−4.1	-	-
A–B	**5**	87.3 (5^a)	87.7	87.1	−0.2	−0.6	+0.4
		88.5 (5^b)			−1.4		−0.8
ring B	**6**	150.2	149.5	149.1	−1.1	−0.4	−0.7
	**7**	126.5	127.6	126.9	+0.4	−0.7	+1.1
	**7^1**	8.8	9.2	9.1	+0.3	−0.1	+0.4
	**8**	146.7	146.0	145.5	−1.2	−0.5	−0.7
	**8^1**	19.3 (8^{1a})	20.8 (8^{1a})	20.7 (8^{1a})	+1.4	−0.1	+1.5
		21.7 (8^{1b})	22.0 (8^{1b})	22.5 (8^{1b})	+0.8	+0.5	+0.3
	**8^2**	39.9 (8^{2a})	39.1 (8^{2a})	40.2 (8^{2a})	+0.3	+1.1	−0.8
		40.7 (8^{2b})	40.1 (8^{2b})	41.8 (8^{2b})	+1.1	+1.7	−0.6
	**8^3**	178.1 (8^{3a})	-	-	-	-	-
		179.2 (8^{3b})	179.4 (8^{3a})	179.9 (8^{3a})	+0.7	+0.5	+0.2
		180.4 (8^{3c})	179.9 (8^{3b})	180.7 (8^{3b})	+0.3	+0.8	−0.5
	**9**	128.1	128.0	127.5	−0.6	−0.5	−0.1
B–C	**10**	111.6	111.9	112.7	+1.1	+0.8	+0.3
ring C	**11**	128.5	128.7	128.1	−0.4	−0.6	+0.2
	**12**	145.8	144.4	144.5	−1.3	+0.1	−1.4
	**12^1**	21.9	21.1	21.1	−0.8	0.0	−0.8
	**12^2**	37.8	38.3	38.9	+1.2	+0.6	+0.5
	**12^3**	179.5	178.1	178.1	−1.4	0.0	−1.4
	**13**	125.0	126.2	126.3	+1.3	+0.1	+1.2
	**13^1**	10.9	11.1	11.2	+0.3	+0.1	+0.2
	**14**	143.1	144.9	145.1	+2.0	+0.2	+1.8
C–D	**15**	94.2 (15^a)	94.3	93.5	−0.7	−0.8	+0.1
		94.9 (15^b)			−1.4		−0.6
ring D	**16**	144.2	144.1	145.1	+0.9	+1.0	−0.1
	**17**	141.1 (17^a)	142.2	142.2	+1.1	0.0	+1.1
		142.3 (17^b)			−0.1		−0.1
	**17^1**	9.6	10.0	9.8	+0.2	−0.2	+0.4
	**18**	133.3	133.9	134.4	+1.1	+0.5	+0.6
	**18^1**	15.3	15.9	16.2	+0.9	+0.3	+0.6
	**18^2**	12.1	12.7	13.2	+1.1	+0.5	+0.6
	**19**	172.2	172.7	172.9	+0.7	+0.2	+0.5

Table S2. [1]H chemical shifts of the carbon-bound protons in PCB chromophore as incorporated in all2699g1, all2699g1-2, and Cph1Δ2 in their respective Pr dark states. The ^{1}H chemical shift differences of PCB carbon-bound protons in the three photoreceptors, $\Delta_{2699g1\text{-}2-2699g1}$, $\Delta_{Cph1\Delta2-2699g1}$, and $\Delta_{Cph1\Delta2-2699g1\text{-}2}$ are reported and illustrated in Figure 3D. The chromophore numbering is according to Figure 1A.

PCB carbon-bound protons		^{1}H chemical shift (ppm) 2699g1	2699g1-2	Cph1Δ2	^{1}H chemical shift difference (ppm) $\Delta_{Cph1\Delta2}$ – 2699g1	$\Delta_{Cph1\Delta2}$ – 2699g1-2	$\Delta_{2699g1\text{-}2}$ – 2699g1
ring A	**2**	1.9 (2^a)	-	-	-	-	-
		2.0 (2^b)	1.8 (2^a)	2.0 (2^a)	**0.0**	**+0.2**	**−0.2**
		2.5 (2^c)	2.4 (2^b)	2.6 (2^b)	**+0.1**	**+0.2**	**−0.1**
	**2^1**	1.7	2.1	2.0	**+0.3**	**−0.1**	**+0.4**
	**3**	2.5 (3^a)	1.9 (3^a)	2.2 (3^a)	**−0.3**	**+0.3**	**−0.6**
		3.1 (3^b)	2.7 (3^b)	2.5 (3^b)	**−0.6**	**−0.2**	**−0.4**
	**3^1**	2.9 (3^{1a})	3.3	3.0	**+0.1**	**−0.3**	**+0.4**
		4.9 (3^{1b})			**−1.9**		**−1.6**
	**3^2**	1.6 (3^{2a})	1.9	1.9	**+0.3**	**0.0**	**+0.3**
		1.9 (3^{2b})			**0.0**		**0.0**
A–B	**5**	6.8 (5^a)	5.4 (5^a)	6.0 (5^a)	**−0.8**	**+0.6**	**−1.4**
		7.5 (5^b)	6.2 (5^b)	6.5 (5^b)	**−1.0**	**+0.3**	**−1.3**
ring B	**7^1**	2.3	2.4	2.4	**+0.1**	**0.0**	**+0.1**
	**8^1**	1.6 (8^{1a})	1.7 (8^{1a})	1.4 (8^{1a})	**−0.2**	**−0.3**	**−0.1**
		3.0 (8^{1b})	2.3 (8^{1b})	2.3 (8^{1b})	**−0.7**	**0.0**	**−0.7**
	**8^2**	3.4	2.8 (8^{2a})	2.3 (8^{2a})	**−1.1**	**−0.5**	**−0.6**
			3.5 (8^{2b})	3.9 (8^{2b})	**+0.5**	**+0.4**	**+0.1**
B–C	**10**	7.9	7.6	7.8	**−0.1**	**+0.2**	**−0.3**
ring C	**12^1**	1.6	2.2	1.0	**−0.6**	**−1.2**	**+0.6**
	**12^2**	3.4	3.5	3.5	**+0.1**	**0.0**	**+0.1**
	**13^1**	2.0	2.1	1.9	**−0.1**	**−0.2**	**+0.1**
C–D	**15**	6.2 (15^a)	5.7	5.7	**−0.5**	**0.0**	**−0.5**
		6.9 (15^b)			**−1.2**		**−1.2**
ring D	**17^1**	2.1	2.3	2.4	**+0.3**	**+0.1**	**+0.2**
	**18^1**	1.8 (18^{1a})	1.4 (18^{1a})	1.7 (18^{1a})	**−0.1**	**+0.3**	**−0.4**
		2.3 (18^{1b})	2.1 (18^{1b})	2.3 (18^{1b})	**0.0**	**+0.2**	**−0.2**
	**18^2**	1.4	1.9	1.5	**+0.1**	**−0.4**	**+0.5**

Table S3. ^{1}H chemical shifts of the protons bound to tetrapyrrole nitrogens [N(21–24)H] in PCB chromophore as incorporated in all2699g1, all2699g1-2, and Cph1Δ2 in their respective Pr dark states. The ^{1}H chemical shift differences of protons bound to tetrapyrrole nitrogens in the three photoreceptors, $\Delta_{2699g1\text{-}2-2699g1}$, $\Delta_{Cph1\Delta2-2699g1}$, and $\Delta_{Cph1\Delta2-2699g1\text{-}2}$ are reported and illustrated in Figure 3D. The chromophore numbering is according to Figure 1A.

N(21–24)H		^{1}H chemical shift (ppm)			^{1}H chemical shift difference (ppm)		
		2699g1	2699g1-2	Cph1Δ2	$\Delta_{Cph1\Delta2\ -\ 2699g1}$	$\Delta_{Cph1\Delta2\ -\ 2699g1\text{-}2}$	$\Delta_{2699g1\text{-}2\ -\ 2699g1}$
ring A	**N21H**	12.3	12.0	12.1	−0.2	+0.1	−0.3
		12.8			−0.7		−0.8
ring B	**N22H**	9.9	10.2	10.7	+0.8	+0.5	+0.3
ring C	**N23H**	10.7	11.6	11.5	+0.8	−0.1	+0.9
ring D	**N24H**	11.6	9.6	10.0	−1.6	+0.4	−2.0

Table S4. Overview of ^{15}N chemical shifts of the PCB chromophore incorporated in all2699g1, all2699g1-2, and Cph1Δ2 in their respective Pr dark states. Published ^{13}C data of Cph1Δ2 [4] were listed for reference. The ^{15}N chemical shift differences of PCB chromophore in the three photoreceptors, $\Delta_{2699g1\text{-}2-2699g1}$, $\Delta_{Cph1\Delta2-2699g1}$, and $\Delta_{Cph1\Delta2-2699g1\text{-}2}$ are reported and illustrated in Figure 4. The chromophore numbering is according to Figure 1A.

Pyrrole nitrogens		^{15}N chemical shift (ppm)			^{15}N chemical shift difference (ppm)		
		2699g1	2699g1-2	Cph1Δ2	$\Delta_{Cph1\Delta2-2699g1}$	$\Delta_{Cph1\Delta2-2699g1\text{-}2}$	$\Delta_{2699g1\text{-}2-2699g1}$
ring A	**N21**	161.2	160.2	160.1	**−1.1**	**−0.1**	**−1.0**
ring B	**N22**	144.7	144.9	147.0	**+2.3**	**+2.1**	**+0.2**
ring C	**N23**	155.8	156.7	158.1	**+2.3**	**+1.4**	**+0.9**
ring D	**N24**	130.9	131.9	131.9	**+1.0**	**0.0**	**+1.0**

Table S5. FWHM line-widths of ^{15}N resonances ($v_{1/2}$) of the PCB chromophore incorporated in all2699g1 and all2699g1-2 in their respective Pr dark states. The ^{15}N experimental line-shapes are simulated by the Voigt function (convolution of a Lorentzian with a Gaussian at an equal ratio). The $v_{1/2}$ values (mean ± standard deviation) are extracted from the Voigt profiles (fitting spectra not shown). $\Delta v_{1/2}$ are listed as all2699g1-2 minus all2699g1. The chromophore numbering is according to Figure 1A.

Pyrrole nitrogens		2699g1		2699g1-2		2699g1-2–2699g1
		δ^N (ppm)	$v_{1/2}$ (FWHM, Hz)	δ^N (ppm)	$v_{1/2}$ (FWHM, Hz)	$\Delta v_{1/2}$ (FWHM, Hz)
ring A	**N21**	161.2	284.2 ± 20.8	160.2	249.1 ± 19.4	**–35.1**
ring B	**N22**	144.7	253.8 ± 16.4	144.9	247.6 ± 22.8	**–6.2**
ring C	**N23**	155.8	315.4 ± 20.3	156.7	235.3 ± 16.5	**–80.1**
ring D	**N24**	130.9	231.2 ± 21.7	131.9	224.7 ± 24.2	**–6.5**

Table S6. Quantitative absorption data of all2699g1-2 variants. UV-vis absorbance maxima (λ_{max}, nm) for the dark state (after far-red irradiation, underlined) and for the photoproduct (after red irradiation) for the PCB adduct of the all2699g1-2 variants and the λ_{max} values for the same samples after denaturation in 8 M urea at pH 2.0. The PCB-loading efficiency of these proteins are listed. The corresponding data for the wild-type all2699g1 and all2699g1-2 are shown as reference.

2699		PCB chromophore		
		Native	Denatured in acidic urea	Loading efficiency (%)
**g1**	**WT**	<u>639</u>/685	<u>663</u>/591	38
**g1-2**	**WT**	<u>638</u>/705	<u>664</u>/596	88
	**W366A**	<u>637</u>/686	<u>666</u>/586	36
	**W366F**	<u>638</u>/690	<u>666</u>/586	22
	**R380I**	<u>639</u>/704	<u>669</u>/595	46
	**S382A**	<u>639</u>/691	<u>665</u>/590	64
	**W386A**	<u>638</u>/689	<u>664</u>/591	41
	**W386F**	<u>639</u>/703	<u>664</u>/590	57
	**R387P**	<u>638</u>/684	<u>666</u>/594	47

Table S7. Primers for the wild type and variants of all2699.

	2699	
**Primer**	**Sequence**	**DNA**
P1	5'-GCCATATGTCACCGACCGCTAAAC-3'	WT (g1)
P2	5'-GCCTCGAGATGACTTTGGGCGAT-3'	
P3	5'-GTCATATGTCACCGACCGCTAAACC-3'	WT (g1-2)
P4	5'-CACTCGAGTTCGTTAAAGGCTTGTACT-3'	
P5	5'-GCATTTAATTTATGGCGTGATGC-3'	S382A (g1-2)
P6	5'-TACACGAGGAAACATTTGCCTCT-3'	
P7	5'-CGTGATGCCAAAAAGTCACAAG-3'	W386A (g1-2)
P8	5'-CGCTAAATTAAATGATACACGAGG-3'	
P9	5'-CGTGATGCCAAAAAGTCACAAG-3'	W386F (g1-2)
P10	5'-GAATAAATTAAATGATACACGAGGA-3'	
P11	5'-GTATCATTTAATTTATGGCGTGATG-3'	R380I (g1-2)
P12	5'-GATAGGAAACATTTGCCTCTGAT-3'	
P13	5'-GATGCCAAAAAGTCACAAGCTCA-3'	R387P (g1-2)
P14	5'-GGGCCATAAATTAAATGATACACG-3'	
P15	5'-GCCGGACGCATTGATCAGGA-3'	W366A (g1-2)
P16	5'-CGCGAGAGTTTCGGTATCTATCTCA-3'	
P17	5'-GCCGGACGCATTGATCAGGA-3'	W366F (g1-2)
P18	5'-GAAGAGAGTTTCGGTATCTATCTCA-3'	

References

1. Heringa, J. Two strategies for sequence comparison: Profile-preprocessed and secondary structure-induced multiple alignment. *Comput. Chem.* **1999**, *23*, 341–364
2. Essen, L.-O.; Mailliet, J.; Hughes, J. The structure of a complete phytochrome sensory module in the Pr ground state. *Proc. Natl. Acad. Sci. USA* **2008**, *105*, 14709–14714.
3. Anders, K.; Daminelli-Widany, G.; Mroginski, M.A.; von Stetten, D.; Essen, L.-O. Structure of the cyanobacterial phytochrome 2 photosensor implies a tryptophan switch for phytochrome signaling. *J. Biol. Chem.* **2013**, *288*, 35714–35725.
4. Stöppler, D.; Song, C.; van Rossum, B.-J.; Geiger, M.-A.; Lang, C.; Mroginski, M.-A.; Jagtap, A. P.; Sigurdsson, S.T.; Matysik, J.; Hughes, J.; Oschkinat, H. Dynamic nuclear polarization provides new insights into chromophore structure in phytochrome photoreceptors. *Angew. Chem. Int. Ed. Engl.* **2016**, *55*, 16017–16020.
5. Rohmer, T.; Lang, C.; Hughes, J.; Essen, L.-O.; Gartner, W.; Matysik, J. Light-induced chromophore activity and signal transduction in phytochromes observed by ^{13}C and ^{15}N magic-angle spinning NMR. *Proc. Natl. Acad. Sci. USA* **2018**, *105*, 15229–15234.
